# High-resolution spatial transcriptomics uncover epidermal-dermal divergences in Merkel cell carcinoma: spatial context reshapes the gene expression landscape

**DOI:** 10.1038/s41388-025-03608-5

**Published:** 2025-10-23

**Authors:** Kuan Cheok Lei, Nalini Srinivas, Mitalee Chandra, Vahan Serobyan, Selma Ugurel, Daniel Hoffmann, Thibault Kervarrec, Weng-Onn Lui, Jürgen C. Becker

**Affiliations:** 1 Translational Skin Cancer Research, German Cancer Consortium (Deutsches Konsortium für Translationale Krebsforschung - DKTK), Partner Site University Medicine Essen, Essen, Germany; 2 German Cancer Research Centre (Deutsches Krebsforschungszentrum - DKFZ), Heidelberg, Germany; 3 Department of Dermatology, University Hospital Essen, Essen, Germany; 4 Department of Dermatology, University Medicine Ostwestphalen-Lippe, Bielefeld, Germany; 5 Bioinformatics and Computational Biophysics, Faculty of Biology, University of Duisburg-Essen, Essen, Germany; 6 Department of Pathology, University Hospital of Tours, Tours, France; 7 Department of Oncology and Pathology, Karolinska Institutet; BioClinicum, Karolinska University Hospital, Solna, Sweden

**Keywords:** Tumour heterogeneity, Sequencing, Cancer microenvironment

## Abstract

Merkel cell carcinoma (MCC) is an aggressive skin cancer with neuroendocrine differentiation marked by high cellular plasticity, often manifesting as rapid therapy resistance. Although the cell-of-origin is presumed to be epithelial, epidermal localization of MCC is rarely observed, largely because in situ MCC is typically an incidental finding. Nevertheless, a subset of MCC tumors exhibits epidermotropism, wherein tumor cells are present in the epidermis. The behavior of cancer cells is profoundly influenced by the tumor microenvironment and interactions with neighboring cells. Notably, the normal counterparts of the cancer’s cell-of-origin have been shown to attenuate tumor aggressiveness. Thus, epidermotropic MCC presents a unique opportunity to explore the potential role of epidermal microenvironment in modulating tumor cell behavior. While the epidermotropic tumor nests share histological resemblance with their dermal counterparts, their transcriptomic profiles remain unexplored. Here, we employed high-definition spatial and single-cell transcriptomics to dissect the gene expression profiles of epidermotropic MCC cells, comparing them to MCC cells in the tumor core and those adjacent to blood vessels. Notably, epidermotropic MCC cells exhibit a transcriptomic signature reminiscent of cutaneous squamous cell carcinoma, characterized by upregulation of genes encoding keratins, S100A proteins, as well as calmodulin-like proteins 3 and 5. Mechanistically, this keratinocytic differentiation is associated with enhanced p63 activity, leading to the upregulation of PERP. Collectively, our study demonstrates that MCC cells can adopt a keratinocytic differentiation program in response to microenvironmental cues, underscoring the remarkable phenotypic plasticity of this malignancy and the importance of the microenvironment for tumor cell characteristics.

## Introduction

Merkel cell carcinoma (MCC) is a rare, aggressive skin cancer with neuroendocrine differentiation usually occurring in the elderly (ref. [1]). MCC cells share features with Merkel cells - the mechanoreceptors found in the basal layer of the epidermis—such as the expression of neuroendocrine markers including chromogranin-A, synaptophysin and cytokeratin 20 (ref. [1]). However, several lines of evidence suggest epithelial cells, either from the hair follicle or the interfollicular skin as the cell-of-origin (ref. [2]). MCC oncogenesis can be virus- or ultraviolet (UV)-driven. Virus-positive MCC is initiated by the clonal integration of Merkel cell polyomavirus (MCPyV) genome into host genome and the constitutive expression of the transforming viral early genes (a.k.a. T-antigens), while UV-mediated DNA mutations are the main driver of virus-negative MCC (ref. [3]).

Histopathological features of MCC include small, monomorphic, round to oval cells with vesicular nucleus and scanty cytoplasm, forming dermal and/or subcutaneous nodules or sheets (ref. [4]). However, epidermotropic MCC cells are occasionally observed. Epidermotropic MCC was first described in 1987 as epidermal Pautrier-like microabscesses, which cooccurred with undifferentiated small cell tumors in the dermis (ref. [5]). D’Agostino et al. reviewed 40 cases of primary MCC tumors, and six of them exhibited epidermotropic tumor growth (ref. [6]). While most reported MCC cases with epidermotropic tumor cells were associated with dermal lesions, Jour et al. described three cases of intraepidermal MCC without dermal components and attributed these as MCC in situ (ref. [7]).

Tumorigenesis is a complex, multistep process initiated by oncogenic mutations or the presence of virally encoded oncogenes, which confer a clonal advantage to normal cells (refs. [7, 8]). However, the transformation of initiated cells into cancer remains a rare event, suggesting the involvement of additional factors beyond genetic alterations. Recent research has highlighted the critical roles of microenvironmental influences and epigenetic modifications in driving early clonal expansion and malignant evolution (ref. [9]). Notably, cancer cells exhibit remarkable plasticity, as demonstrated by their ability to revert to a more normal phenotype when placed in a non-tumorigenic microenvironment (refs. [10, 11]). This underscores the pivotal role of the tumor microenvironment (TME) in cancer cell characteristics (ref. [12]).

Epidermotropic MCC presents a unique model to study how the local microenvironment influences tumor cell behavior (refs. [5–7, 13]). Despite this potential, there is a notable lack of transcriptomic studies characterizing the gene expression profiles of epidermotropic MCC cells, likely due to technical challenges in isolating and analyzing these rare cell populations. To address this gap, we employed high-definition spatial transcriptomics combined with advanced image segmentation techniques. This approach enabled us to generate spatially resolved, single-cell-level gene expression data, revealing a distinct transcriptomic signature that differentiates epidermotropic MCC cells from their dermal counterparts. These findings provide new insights into the molecular mechanisms underlying MCC behavior and its interaction with the epidermal microenvironment.

## Materials and methods

Detailed methods of spatial library preparation, analyses of cell segmentation data, analyses on single-cell RNA sequencing data, quantification of *PERP*, *TAP53* and *ΔNP63* in MCC cell lines, and *TAP53* overexpression experiment with WaGa cell lines can be found in Supplementary Methods from Supplementary Information.

### Tumor tissue

Four primary MCC with both dermal and epidermal compartments from the archive of Translational Skin Cancer Research, German Cancer Consortium (DKTK), Essen, Germany, in collaboration with the University Hospital Essen, where patients with MCC were enrolled and taken biopsies, were chosen for analysis. The study was approved by the Ethics Committee of the University of Duisburg-Essen (11-4715; 17-7538-BO) and was conducted in accordance with the Declaration of Helsinki—Ethical Principles for Medical Research Involving Human Subjects. Written informed consent was obtained from all subjects involved in the study. Patients and tumor characteristics, including MCPyV status, are provided in Table 1. Immunohistochemistry staining for CK20 was performed to confirm the presence of epidermal MCC cells using the mouse monoclonal antibody clone D9Z1Z (Cell Signaling, Massachusetts, USA) at a dilution of 1:50. Antigen retrieval was carried out using Tris-EDTA buffer (pH 9.0) in a pressure cooker. High-resolution whole slide scans of hematoxylin & eosin (H&E) stained sections used to align spatial transcriptomics data are depicted in Supplementary Figs. S1–4.

**Table 1:** Patient and tumor characteristics.

Sample #	Age	Sex	Localization	Type	MCPyV status^a^
1	82	M	NA	Primary	Negative
2	84	M	Left corner of the eye	Primary	Positive
3	72	M	Right corner of the eye	Primary	Negative
4	84	F	Left arm	Primary	Negative

*M* male, *F* female; *NA* not available.

^a^MCPyV status was assessed by qPCR as previously described by Fan et al. (2020) 10.1016/j.jid.2019.06.135.

### Cell lines and culture conditions

All MCC cell lines (WaGa, MKL-1, MKL-2, UM-MCC002, UM-MCC005, UM-MCC0034) have been described before (ref. [14]). Cells were maintained at 37 °C in a humidified atmosphere containing 5% CO₂, in RPMI 1640 medium with Glutamine and 2 g NaHCO2 (PAN-Biotech, Cat. No. P04-16500) supplemented with 10% fetal bovine serum (PAN-Biotech, Cat. No. P40-37500).

## Results

### High-definition spatial sequencing and cell segmentation

**Fig. 1:**
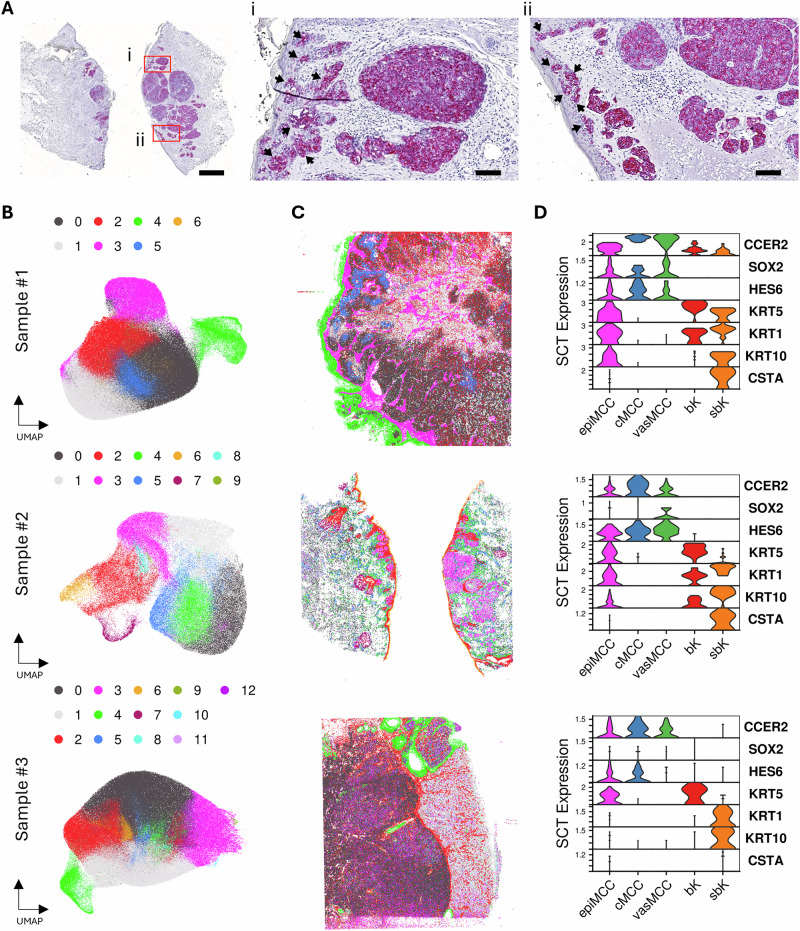
Spatial transcriptomic profiling and single-cell analysis of primary MCC lesions exhibiting epidermotropism. **A** Immunohistochemistry staining for Cytokeratin 20 in sample #2 in 20× magnification with 1 mm scale bar at the bottom right. High power fields (200×, i and ii) highlights epidermotropic MCC cells forming intraepidermal nests, as indicated by arrows, 100 μm scale bars are shown at the bottom right. **B** UMAP dimensionality reduction of segmented single-cell data inferred from spatial transcriptomic data, colors represent distinct transcriptional clusters. **C** Spatial mapping of segmented single-cell data with cells color-coded as defined in (**B**). **D** Normalized expression of representative gene markers in histomorphologically annotated cell populations: epiMCC epidermal MCC, cMCC tumor core MCC, vasMCC perivascular MCC, bK basal keratinocytes, and sbK suprabasal keratinocytes.

Four MCC primary tumor samples exhibiting epidermotropism, as demonstrated by the presence of CK20-positive MCC cells in the epidermal compartment (Fig. 1A), were included in the study. Patient and tumor details are listed in Table 1. The median age of patients was 83 years, 2 of the tumors were localized in the face, 3 of them were MCPyV-negative (VN) and one MCPyV-positive (VP). The samples were processed for Visium Spatial HD sequencing as described in “Materials and Methods” (Supplementary Figs. S1–4). Unfortunately, in sample #3, the epidermotropic MCC cells located in the interfollicular epidermis were not captured within the sequenced region, thus the analysis of epidermotropic MCC cells in this sample was limited to those localized within the follicular infundibulum. After sequencing, 545,086,555 reads were obtained for sample #1 (VN), 375,522,853 reads for sample #2 (VP), 402,504,271 reads for sample #3 (follicular infundibulum intraepidermal MCC cells, VN) and 565,505,414 reads for sample #4 (VN), with an average sequencing coverage of 940.1 reads, 970.8 reads, 730.1 reads and 932.5 reads per 8 μm bin, respectively. Data analysis using the 10X Space Ranger pipeline generated graph-based unsupervised clusters of 8 μm count matrices, and the spatial localization of these clusters corresponded to the histological patterns observed in each sample (Supplementary Figs. S6–9).

To harness the sub-cellular resolution capabilities of HD spatial sequencing, we employed cell segmentation using bin2cell (ref. [15, 16]). This approach enabled the reconstruction of cell-level gene expression profiles by utilizing the 2 μm resolution of the Visium Spatial HD sequencing output. After filtering the segmented cell sets as specified in the “Materials and methods” section, 351,149 cells were retained for sample #1, 117,976 cells for sample #2, 363,701 cells for sample #3 and 353,424 cells for sample #4. As the total number of reads was relatively similar to that of single-cell RNA sequencing while targeting 10- to 30-fold more cells, the segmented cells had consequently shallower coverage than those from single-cell RNA sequencing (Supplementary Fig. S10). Specifically, individual cells in sample #1 had an average of 421.9 expressed genes and 650.7 UMIs, for sample #2, 150.3 expressed genes and 219.7 UMIs, for sample #3, 206.9 expressed genes and 287.0 UMIs, and for sample #4, 149.0 expressed genes and 184.1 UMIs (Supplementary Table 1). We considered sample #4 to be insufficiently sequenced based on the ranges of expressed genes and UMI counts in segmented cell data, thus excluded it from downstream analysis with segmented cell sets. Although the number of genes detected in the individual cells provided a lower coverage of the expression profile than with the 8 × 8 µm^2^ approach, the spatial localization of the back projection of the clustering of single-cell level corresponded to the patterns observed with the coarser approach (Fig. 1B, C). Thus, the spatial transcriptomics-based gene expression profile at single-cell level is sufficiently granular to enable precise identification of the respective cell type and functional state.

For further analysis, we use the morphological information on phenotype and localization in the H&E and CK20-stained sections to select cells of interest (refs. [17, 18]). We employed the interactive visualization with Napari provided by python package Squidpy v.1.2.2, to superimpose segmented cell coordinates from bin2cell output onto H&E image for individual cell selection (see “Materials and methods”). The H&E images were inspected visually by two independent observers, and only cells of interest with a corresponding unambiguous segmented cell coordinate were included. For epidermal MCC cells (epiMCC; *n*_1_ = 49, *n*_2_ = 80 and *n*_3_ = 62), we targeted epidermal tumor cell nests that were entirely embedded within the epidermis; core MCC cells (cMCC, *n*_1_ = 68, *n*_2_ = 62 and *n*_3_ = 69) were located within the dermal tumor nodules. As a subgroup of the latter, we also selected cells near vascular structures (vasMCC; *n*_1_ = 51, *n*_2_ = 73 and *n*_3_ = 55). For comparison, we picked a number of basal (bK; *n* = 39) and suprabasal (stratum spinosum; sbK; *n* = 40) keratinocytes. Basal keratinocytes were selected from the basal monolayer of the epidermis and suprabasal keratinocytes were selected within stratum spinosum without nearby tumor structures. We confirmed that MCC nature of the selected cells by expression of the established marker genes *CCER2* (coiled-coil glutamate-rich protein 2), *SOX2* (SRY-box transcription factor 2), and *HES6* (Hes family BHLH transcription factor 6) (Fig. 1D) (refs. [19, 20]). *KRT5* (keratin 5), *KRT1* (keratin 1), *KRT10* (keratin 10) and *CSTA* (cystatin A) were used as marker genes for keratinocytes. *CSTA* was more abundantly expressed in suprabasal keratinocytes compared with basal keratinocytes, along with other suprabasal markers such as *KRT1* and *KRT10*, consistent with its established role as a key precursor in the formation of the cornified envelope during corneocyte development (ref. [21]). This expression pattern further validated the specificity of our cell segmentation and manual selection processes.

### Epidermal MCC shares transcriptomic profile with SCC

**Fig. 2:**
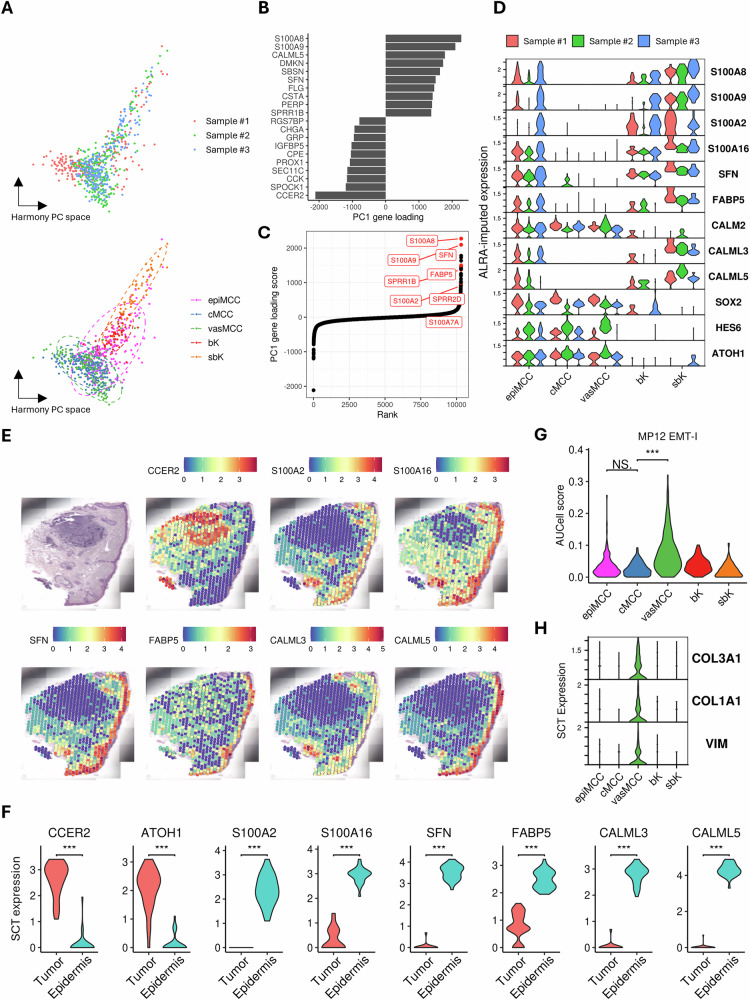
Transcriptomic convergence between epidermotropic MCC, keratinocytes, and cutaneous squamous cell carcinoma. **A** Harmony-corrected principal component analysis (PCA) of histomorphologically selected single-cell data, annotated by sample number (upper) and by cell type [lower; epidermal MCC (epiMCC), tumor core MCC (cMCC), perivascular MCC (vasMCC), basal keratinocytes (bK), and suprabasal keratinocytes (sbK)]. Ellipses in the lower panel represent the 95% confidence interval for the multivariate t-distribution for each cell type cluster. Explained variance for Principal Component (PC)1: 2.3% and PC2: 2.9%. **B** Top contributing genes driving variation PC1. **C** Genes are ranked according to their contribution to PC1, and markers associated with cutaneous squamous cell carcinoma (cSCC) are highlighted in red. **D** Imputed expression levels of cSCC markers as well as epidermal genes *CALML3* and *CALML5* in the combined dataset of histomorphologically selected cells from the three samples. **E** H&E staining and spatially resolved gene expression (Spot-based Visium) of an MCC tumor without epidermotropism. Expression of cSCC markers (*S100A2*, *S100A16*, *SFN*, *FABP5*) and epithelial genes *CALML3*/*CALML5* is restricted to the epidermis and hair follicles and absent in the *CCER2*+ tumor area. Color bars represent normalized expression. **F** Spatially resolved, normalized expression in the tumor depicted in (E) of MCC markers (*CCER2*, *ATOH1*), cSCC markers (*S100A2*, *S100A16*, *SFN*, *FABP5*) and *CALML3*/*CALML5* dichotomized in tumor and epidermal areas. **G** AUCell scoring of the epithelial-mesenchymal transition (EMT)-related tumor transcriptional metaprogram (MP) 12 on different cell types in the combined dataset of histomorphologically selected cells from the three MCC with epidermotropism. **H** Violin plots depict the normalized expression of *COL3A1, COL1A1*, and *VIM* encapsulated within MP12.epiMCC epidermal MCC, cMCC core MCC, vasMCC perivascular MCC, bK basal cell, sbK suprabasal keratinocyte. ***P < 0.001, **P < 0.01, *P < 0.05, NS not significant by Wilcoxon *t* test, unadjusted.

Due to the limited number of selected cells and the sparse gene expression profiles resulting from cell segmentation, we adopted an alternative approach to conventional differential gene expression analysis, which typically relies on non-parametric statistical tests. Specifically, we collected the cells from the three samples in a unified dataset and applied principal component analysis (PCA). This allowed us to identify and analyze the gene weights associated with the principal components of interest, providing a more global analysis of the data. To reduce the effect of keratin expressions on dimensionality reduction, keratin genes were excluded from PCA. The first PC differentiated both basal and suprabasal keratinocytes as well as epidermal MCC cells from core MCC cells. A 95% confidence ellipse, based on a multivariate t-distribution, was calculated for each cell type cluster in the reduced-dimensional space. The cluster corresponding to epidermotropic MCC cells was positioned between the tumor core MCC and keratinocyte clusters (Fig. 2A). Notably, epidermal MCC cells exhibited greater similarity to basal keratinocytes than to suprabasal keratinocytes. Top loading genes of the first PC in the combined dataset included *SFN* encoding stratifin, calcium-binding S100 protein genes *S100A8* and *S100A9*, calmodulin-like protein genes *CALML3* and *CALML5* (Fig. 2B, Supplementary Table 2). Interestingly, the top PC genes which distinguished epidermal MCC cells from core MCC cells resembled the gene markers of cutaneous squamous cell carcinoma (cSCC). Hence, we compared the gene loading of the first PC with the gene markers of cSCC reported by Yan et al. (ref. [22]). The result showed a significant enrichment of cSCC markers such as *S100A2*, *FABP5*, *KRT6A/B* and *SPRR1B* (Fig. 2C). Imputed gene expression analysis using the zero-preserving imputation algorithm ALRA (ref. [23]) revealed prominent expression of cSCC markers such as S100A genes and *CALML3/5* in epidermal MCC cells but not in core MCC cells (Fig. 2D).

S100 proteins and calmodulins are calcium-binding proteins that function as intracellular calcium carriers (e.g., S100 proteins) and sensors (e.g., calmodulins). Both are expressed in normal epithelial cells. The similar regulation of these calcium-signaling genes in epidermal MCC cells and basal and suprabasal keratinocytes suggests that they could exhibit similar phenotypes in an epidermal microenvironment. (ref. [24]). To investigate this hypothesis, we conducted spatial transcriptomics on a dermal MCC sample without epidermal involvement, serving as a control (Supplementary Fig. S5). The MCC marker *CCER2* was strongly expressed in the tumor, while epidermal MCC-associated genes, such as *S100A2* and *CALML3/5*, were largely absent in MCC cells, but highly expressed in the epidermis. This observation was statistically validated using the Wilcoxon rank-sum test on 30 selected spots from both tumor and epidermal regions (Fig. 2E, F). These findings suggest plasticity of MCC cells is dependent on the spatial context, i.e., within the epidermis or dermis. In addition, we scored the cells using AUCell on tumor transcriptional metaprograms, to evaluate the transcriptional states of each MCC subtypes (ref. [25]). Notably, gene set enrichment analysis of metaprogram 12 (MP12) identifies elevated epithelial-mesenchymal transition (EMT) activity in perivascular MCC (vasMCC) compared with other core MCC cells situated further from blood vessels (cMCC). (Fig. 2G). Specific gene expression analysis confirmed upregulation of EMT-related genes, including COL3A1, COL1A1, and *VIM* in vasMCC cells (Fig. 2H). These results underscore the crucial influence of spatial localization in modulating the phenotype of MCC cells and suggest microenvironment-driven plasticity.

### Cross-validating spatial transcriptomics results with single-cell transcriptomics

To validate the results from HD spatial transcriptomics, we reanalyzed the single-cell RNA sequencing (scRNAseq) data from Das et al. (ref. [26]). Among the 11 MCC samples from 9 patients, 5 samples were primary tumors; thus, from the original dataset of 79,137 cells, we only retained the 30,153 cells specifically originating from primary tumors. Cells were annotated based on the normalized expression of target genes identified through HD spatial transcriptomics. The following criteria were used to define distinct MCC subpopulations: EpiMCC—non-zero expression of CCER2 and KRT5, along with the expression of either CALML3 or CALML5; cMCC—co-expression of CCER2 and KRT20, with no detectable expression of COL3A1, COL1A1, or KRT5; and vasMCC—non-zero expression of both CCER2 and KRT20, along with the expression of either COL3A1 or COL1A1. Applying these criteria, we identified 88 epiMCC, 8551 cMCC, and 598 vasMCC cells within the scRNA-seq data. To ensure comparability across groups and avoid dominance of more abundant cell types in PCA, larger cell populations were subsampled to match the size of the smaller groups.

**Fig. 3:**
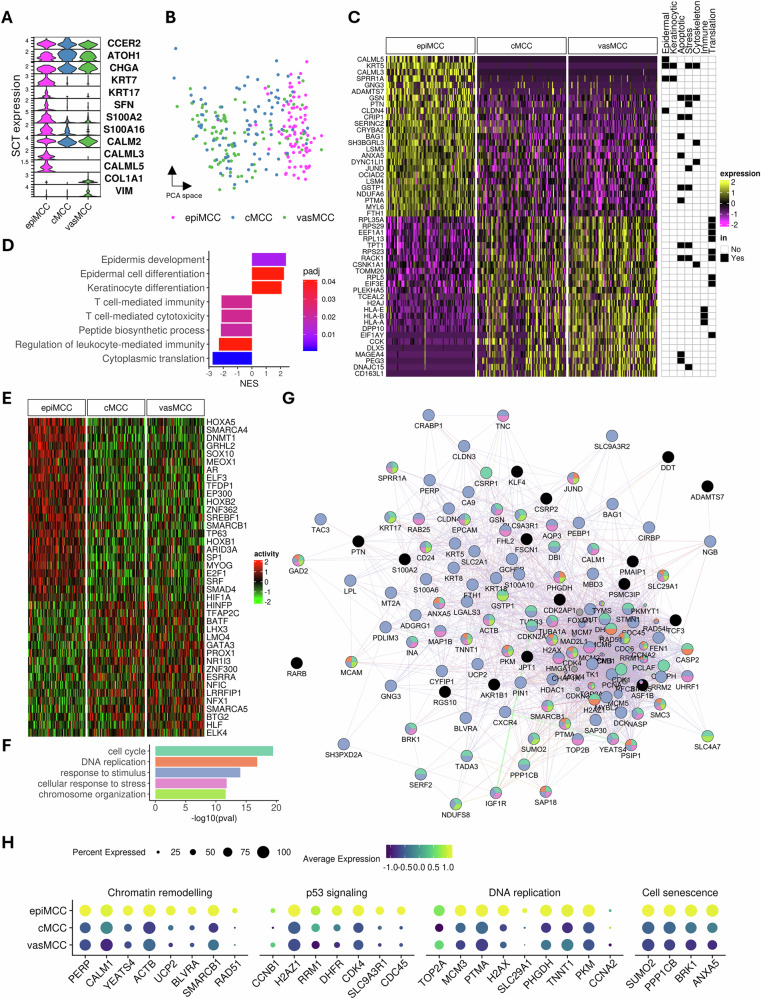
Single-cell transcriptomic validation of epidermotropic MCC phenotypes. **A** Violin plot depicting normalized gene expression of *CCER2, ATOH1, CHGA, KRT7, SNF, S100A2, S100A16, FABP5, CALM2, CALML3, CALML5, COLA1*, and *VIM* in epiMCC, cMCC and vasMCC cells identified via stringent filtering criteria in the independent single-cell RNA sequencing datasets (GSE226438). **B** Harmony-corrected principal component analysis (PCA) of epiMCC, cMCC and vasMCC cells from (**A**). Explained variance for PC1: 5.8%; PC2: 5.7%. **C** Heatmap of the top differentially expressed genes (filtered by significance and ranked by fold change) between epiMCC, cMCC, and vasMCC. Among the top differentially expressed genes are epidermal, keratinocytic, stress and immune evasion-associated markers. **D** Gene set enrichment analysis (GSEA) with GO biological processes of genes differentially expressed in epiMCC compared to cMCC. Color represents adjusted *p* values. **E** Heatmap of transcription factor (TF) regulons with enhanced activity in epiMCC, prioritized by differential regulon specificity scores. **F** Gene Ontology (GO) analysis of biological processes enriched among 114 epiMCC-upregulated genes co-regulated by TFs in (**E**). **G** Functional interaction network of target genes in (**F**), color-coded by associated GO terms as in (**F**). **H** Dot plots of representative genes upregulated in epiMCC from key pathways, underscoring keratinocytic reprogramming. Dot size represents the proportion of cells in which each gene was expressed, color the average expression. epiMCC epidermotropic MCC, cMCC core MCC, vasMCC perivascular MCC.

Expression of neuroendocrine markers such as *ATOH1* and *CHGA* was abundant across all three MCC cell subtypes, and the specificity of selection was demonstrated by expressions of epithelial-related genes such as *SFN* in epiMCC and EMT-related genes such as *VIM* in vasMCC cells (Fig. 3A). PCA demonstrated a distribution of phenotypes along the first PC, with epiMCC cells on one end and the majority of vasMCC cells on the other end (Fig. 3B). Differential gene expression analysis identified several epithelial-related genes upregulated in epiMCC compared with cMCC and vasMCC cells, such as *SPRR1A* encoding cornifin-A and *CLDN4* encoding claudin 4; moreover, it revealed a handful of apoptosis- and stress-related genes, such as *JUND* encoding JunD, *GSN* encoding gelsolin, *BAG1* encoding BCL2-associated athanogene 1 and *GSTP1* encoding glutathione S-transferase P1, which protect the cells from p53-mediated cell death (refs. [27–30]) (Fig. 3C, Supplementary Table 3). Gene set enrichment analysis for differentially expressed genes between epiMCC and combined group of cMCC and vasMCC identified enhanced epithelial development and keratinocyte differentiation in epiMCC cells, whereas cMCC showed signs of immune activation and proliferation (Fig. 3D). This finding was consistent with observations from HD spatial transcriptomic cell segmentation data.

### Epithelial differentiation regulons are active in epidermal MCC cells

To further investigate the impact of the spatial context on the transcriptional activities of MCC cells, we performed transcription factor activity inference. The activity of a transcription factor is reflected in the regulation of its target gene network (regulon), which includes genes that are either activated or repressed. We observed higher activities of E2F1 and TFDP1 in epiMCC cells, both known activators of cell proliferation that are commonly overexpressed in MCC cells (Fig. 3E, Supplementary Table 4). Interestingly, TP63 coding for keratinocyte proliferation regulator p63 (ref. [31]) was also enhanced in epiMCC (Fig. 3E, Supplementary Table 4). This observation is in line with keratinocytic differentiation of epiMCC cells observed in the differential gene expression analysis. To provide a clearer overview of the upregulated regulons in epiMCC cells, we identified 114 differentially expressed genes that were both upregulated in epiMCC cells and regulated by the enhanced transcription factors. Functional analysis of these target genes revealed a network of 134 genes, with enrichment in cell cycle regulation (53 genes) and DNA replication (24 genes) as the top two functional terms in the GO biological process category. Furthermore, the largest functional group was the stress response, encompassing 103 genes (Fig. 3F, G, Supplementary Table 5). The enriched terms can be categorized into four consensus pathways: chromatin remodeling, p53 signaling, DNA replication, and cell senescence. Notable genes upregulated in epiMCC cells included minichromosome maintenance genes (*MCM3*), cyclin genes (*CCNA2* and *CCNB1*), and the cyclin-dependent kinase gene (*CDK4*) (Fig. 3H). These genes play essential roles in cell cycle regulation and DNA replication, supporting the plausibility of the identified pathways. Given the rarity of epiMCC cells in both spatial and single-cell RNA sequencing data, the observed enhancement in cell cycle regulation does not necessarily indicate uncontrolled proliferation. Instead, these findings suggest a tightly regulated mechanism of cell cycle control, reinforcing the role of these pathways in maintaining genomic stability rather than driving tumorigenic growth (ref. [32]).

### PERP is enhanced as a result of upregulated p63 activity

**Fig. 4:**
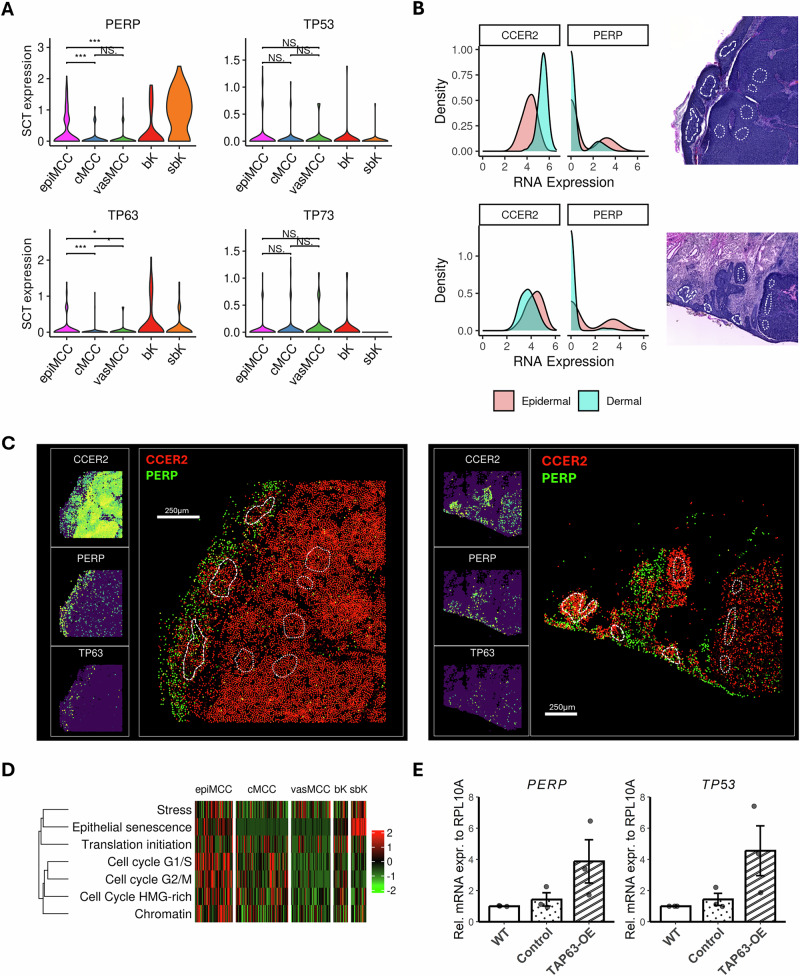
PERP upregulation defines the keratinocytic differentiation program in epidermotropic MCC cells. **A** Violin plots of normalized expression of *PERP, TP53, TP63, and TP73* in histomorphologically selected epiMCC, cMCC, vasMCC, bK and sbK cells in samples #1, #2, and #3. **B** Density plots comparing expressions of the MCC lineage marker gene *CCER2* and the epidermal differentiation gene *PERP* in epiMCC regions (dashed lines, intraepidermal) and cMCC regions (dotted lines, dermal). **C** Spatially resolved two-color gene co-expression visualization of *CCER2* (MCC identity) and *PERP* (epidermal reprogramming) in samples #1 (left) and #2 (right). **D** Heatmap depicting the activity of selected tumor transcriptional metaprograms (MP) computed with AUCell using normalized gene expression of the same selected cells as (**A**). **E** Relative mRNA expression of *PERP* and *TP53* in wild type (WT), mock-transfected (control), and *TAP63*-overexpressing (TAP63-OE) WaGa MCC cells. Gene expression level was normalized to wild-type WaGa cells. Values are in mean ± S.E.M. of 3 independent biological replicates; average Cq values of target genes are shown in Supplementary Table S7. epiMCC epidermotropic MCC, cMCC core MCC, vasMCC core MCC near blood vessel, bK basal cell, sbK keratinocyte. ***P < 0.001, **P < 0.01, *P < 0.05, NS not significant by Wilcoxon *t* test, unadjusted.

Intriguingly, one of the genes associated with chromatin remodeling was *PERP* (encoding the protein P53 apoptosis effector related to PMP-22). PERP is directly regulated by p53 family members (i.e., p53 in response to cellular stress or DNA damage and p63 in epithelial development and maintenance) and is thus implicated as a tumor suppressor in human cancers (ref. [33, 34]). Beyond its role in apoptosis, PERP is a key component of desmosomes—structures that facilitate cell adhesion in epithelial tissues (ref. [35]). *PERP* was significantly upregulated in epiMCC cells in the segmented cell data (Fig. 4A). Next, we revisited the H&E images and selected regions enriched for epiMCC and cMCC cells instead of individual cells to annotate segmented cells from the selected areas. Cells lacking expression of the tumor marker gene *CCER2* expression were excluded to ensure that the analyzed cells comprised tumor cells rather than keratinocytes. While epiMCC and cMCC cell areas exhibited comparable *CCER2* expression levels, *PERP* expression was markedly lower in cMCC cells than in epiMCC cells (Fig. 4B). Visualizing the mRNA expressions of *CCER2* and *PERP* in spatial coordinates, we observed that the co-expression of these genes was more common in the epidermal compartment compared to the tumor core (Fig. 4C). Interestingly, in sample #2, a small core MCC nodule located near a hair follicle did not exhibit increased *PERP* expression, despite its close proximity to *PERP*-high hair follicle cells. This finding stood in stark contrast with sample #3, where *PERP* upregulation was observed in follicular infundibulum epiMCC cells. Additionally, analysis of tumor transcriptional metaprogram activities using normalized gene expression data revealed enhanced activation of epithelial senescence and cell cycle regulatory programs in epiMCC cells compared to cMCC cells (Fig. 4D). These results demonstrate the phenotypic plasticity of MCC cells, which is influenced by direct interactions with the surrounding microenvironment.

### Induction of PERP by experimental TAp63 overexpression

We hypothesized that the upregulated *PERP* expression was the result of transcriptomic shift towards MCC cell-of-origin, which was regulated by epidermal development regulator p63. p63 exists in two main classes of isoforms: the transactivation (TA) isoforms (TAp63), which contain an N-terminal transactivation domain, are primarily expressed in stratified epithelial cells and initiate keratinocyte differentiation during embryogenesis; and the ΔN isoforms (ΔNp63), which lack this domain, promote terminal differentiation of keratinocytes and are predominantly found in the mature epidermis (ref. [36]). Both of p63 isoform classes can transactivate *PERP* transcription, although ΔNp63 induces approximately half the *PERP* expression level compared with TAp63 (ref. [37]). We first assessed the relative expression of mRNA coding for *PERP*, the 2 *TP63* isoforms (*TAP63* and *ΔNP63) and TP53* in six MCC cell lines (WaGa, MKL-1, MKL-2, UM-MCC002, UM-MCC005, UM-MCC034) by qPCR. *TAP63* was not detected in any MCC cell lines, whereas *ΔNP63* was expressed (Supplementary Fig. S11A, Supplementary Table S6). Interestingly, *PERP* expression was higher in virus-positive cell lines than in the virus-negative cell line (UM-MCC034). Given the lack of *TAP63* expression in MCC cell lines and considering the established role of TAp63 in initiating keratinocytic differentiation, we speculated that elevated *PERP* expression in epiMCC would be most likely induced by TAp63-upregulated p63 activities. To test this, we overexpressed *TAP63* in the MCC cell line WaGa and quantified the relative expression levels of *PERP, ΔNP63* and *TP53* (Supplementary Fig. S11B, D). Upon *TAP63* overexpression, *ΔNP63* showed a drastic change in expression level, with 2 out of 3 replicates (66.7%) having reduced expression compared with wild-type (Supplementary Fig. S11E), demonstrating an inversely correlated expression of the two isoforms as described previously (ref. [38]). Notably, overexpression of *TAP63* in WaGa cells led to increased *PERP* expression (Fig. 4E, Supplementary Fig. S11D). Interestingly, TP53 was also upregulated in these cells following *TAP63* overexpression. A notion consistent with previous reports (ref. [39]). These findings suggest that PERP may function as a tumor suppressor downstream of p53 regulation, in addition to its role in epidermal differentiation (ref. [34]).

## Discussion

The spatial context of cancer cells—dictated by their interactions with neighboring cells and the extracellular matrix (ECM)—plays a pivotal role in modulating their behavior. These interactions are critical drivers of cancer progression, metastasis, and therapeutic resistance. Intriguingly, cancer cells can revert to a less aggressive phenotype when reintroduced into a normal tissue environment. In the context of Merkel cell carcinoma (MCC), the phenomenon of epidermotropism—characterized by the infiltration of MCC cells into the epidermis—provides a unique model to explore the impact of the epidermal microenvironment on tumor cell behavior. Although epidermotropism in MCC represents a rare growth pattern, distinct from the more prevalent dermal involvement, it offers valuable insights into the plasticity of tumor cell phenotypes.

Leveraging recent technological advances in high-resolution spatial transcriptomics, we took advantage of such cases to establish the transcriptomic profiles of MCC cells located in the epidermis and compared them to their dermal counterparts within the same tumor. However, when utilizing Visium Spatial HD sequencing data to generate spatially resolved gene expression profiles at single-cell resolution, we realized that while the native outputs offered finer resolution and greater coverage than the spot-based Visium spatial sequencing, the rectangular binning approach suggested by the manufacturer proved suboptimal for downstream applications. To overcome this limitation, we tailored an analysis workflow that combined Visium Spatial HD data with image segmentation algorithms to resolve cell-based gene expressions from spatial sequencing data without the need for cell deconvolution. The approach revealed that epidermal MCC cells exhibited upregulated expression of keratins, S100 proteins, and calmodulin-like proteins 3 and 5. Notably, these cells displayed heightened cell cycle activity, closely resembling cSCC cells. However, the upregulation of *PERP* in epiMCC cells also suggested a potential suppressive effect on tumor growth, underscoring the intricate interplay between MCC cells and the epidermal microenvironment.

Interestingly, the top PC genes which distinguished epiMCC cells from cMCC cells resembled the gene markers of cSCC. The association between SCC and MCC, particularly virus-negative MCC, has been previously described (ref. [40]). Harms et al. studied seven cases of intraepidermal MCC-associated cSCC in situ with a dermal MCC component, and demonstrated that cSCC in situ associated with MCC exhibited upregulation of both epidermal markers, such as *KRT6A* and *KRT6B*, and intermediate levels of neuroendocrine markers like *SYP* and *DACH1*, implying clonal relatedness between virus-negative MCC and associated cSCC in situ (ref. [41]). Furthermore, Kervarrec et al. performed whole-exome sequencing with four cases of cSCC in situ with invasive MCC, and observed 12.8–92.0% of the somatic variants were shared by both cSCC in situ and MCC components in the same specimen (ref. [42]).

Both p63 and PERP play critical roles in the proper differentiation of keratinocytes. p63 serves as the master regulator, orchestrating cell proliferation and differentiation, while PERP contributes to these processes by maintaining desmosome formation and cell–cell adhesion, which are critical for epithelial integrity. In the epidermal MCC, p63’s transcriptional activity was notably enhanced, suggesting that p63-mediated transactivation drives PERP upregulation, thereby promoting a keratinocyte-like phenotype (ref. [37]). Our in vitro findings provide an additional line of evidence that the TAp63–PERP axis may play a tumor-suppressive role MCC. While endogenous TAp63 expression was not detected in the MCC cell lines analyzed, forced overexpression of *TAP63* led to increased transcript levels of both PERP and TP53, which is consistent with previous reports demonstrating the involvement of TAp63 in the activation of tumor suppressor pathways (ref. [34]). While both TAp63 and ΔNp63 isoforms can transactivate PERP, TAp63 does so more efficiently (ref. [37], emphasizing the unique significance of TAp63 in this regulatory network. Functionally, TAp63 acts primarily as a tumor suppressor by inducing senescence, regulating apoptosis, and inhibiting oncogenic signaling pathways (ref. [43]). Overall, activation of the TAp63–PERP pathway may contribute to tumor suppression in MCC by promoting p53-mediated effects and reinforcing epidermal differentiation programs. However, it is important to note that p63 has been consistently associated with reduced overall survival and disease-free survival in MCC patients, highlighting its complex and context-dependent role in tumor biology (refs. [44, 45]).

Further evidence supporting keratinocytic differentiation in epiMCC cells is the expression of calmodulin-like protein genes *CALML3* and *CALML5*, in contrast to cMCC cells, particularly those in the perivascular niche, which predominantly express *CALM2*. CALML3 and CALML5 play essential roles in maintaining epithelial functions and integrity, whereas CALM2 has been implicated in promoting aggressive cancer phenotypes, including invasion, metastasis and immune evasion (ref. [46]). CALML3 primarily regulates calcium homeostasis and protects against oxidative stress-induced damage, CALML5 acts as a tumor suppressor by promoting cell adhesion and preventing excessive motility, while CALM2 modulates key cancer-related pathways such as PI3K/AKT, MAPK/ERK, and JAK/STAT, promoting cell survival and proliferation (refs. [46, 47]). While the exact mechanisms remain speculative, CALML3 and CALML5 likely modulate CALM2 through structural competition, calcium buffering, or indirect pathway regulation. Although itself not a direct target of p63, *CALML5* is upregulated by ZNF750 and KLF4 via the p63-ZNF750-KLF4-CALML5 activation axis (ref. [47–49]). Interestingly, *CALML5* is also predicted to be directly regulated by p63 via potential p63-binding sites in the promoter regions, though there were discordant results from the literature (ref. [48, 50]).

Direct and indirect interactions between epiMCC cells and keratinocytes may influence the transcriptomic profile of MCC cells. We previously demonstrated that knockdown of MCPyV T-antigens in MCC cells triggers neuronal differentiation, a process characterized by the loss of stem-like properties, reactivation of the RB pathway, and upregulation of genes associated with neurogenesis (ref. [20]). This differentiation was further modulated by key transcriptional regulators. Notably, these effects were contingent upon the presence of normal keratinocytes, highlighting a critical role for the TME in mediating this phenotypic shift. Nearby keratinocytes may render MCC cells less aggressive through processes such as inducing senescence, preserving epithelial integrity, inhibiting EMT, modulating signaling pathways, and enhancing immune surveillance (refs. [51, 52]). These findings underscore the critical role of the TME in cancer progression and suggest therapeutic strategies that exploit these natural defenses, particularly during the early stages of carcinogenesis.

Collectively, our findings illuminate the profound influence of the epidermal microenvironment in driving keratinocytic differentiation of MCC cells through p63/PERP axis activation, reinforcing the concept that phenotypic plasticity in MCC is dynamically shaped by stromal interactions. This study provides molecular insights into how epidermotropism—a histopathological hallmark of a subset of MCC tumors—may reflect a differentiation state modulated by keratinocyte-derived cues. Importantly, these insights suggest that keratinocytic differentiation markers, such as PERP, could serve as prognostic indicators of tumor behavior, while therapeutic strategies targeting p63-mediated pathways may offer novel avenues to mitigate MCC plasticity and treatment resistance.

## Supplementary information

Legends of Supplementary Figures and Tables

Supplementary Information

Supplementary Table S1

Supplementary Table S2

Supplementary Table S3

Supplementary Table S4

Supplementary Table S5

Supplementary Table S6

Supplementary Table S7

Supplementary Table S8

## Data Availability

Raw data used in this study are deposited in European Genome-Phenome Archive under accession number EGAS00001008157 and are available upon request to the corresponding author, in adherence to the General Data Protection Regulation in the European Union. Processed data for histomorphologically selected cells as well as selected cells from validation single-cell data can be retrieved at 10.6084/m9.figshare.28495241.

## Code availability

The codes for running the cell segmentation, as well as performing the bioinformatic analyses, can be found in the GitLab repository hosted by the University of Duisburg-Essen https://git.uni-due.de/adk625f/epimcc.
