# Supplementary material for: High-resolution spatial transcriptomics uncover epidermal-dermal divergences in Merkel cell carcinoma: spatial context reshapes the gene expression landscape
Source: Oncogene. 2025 Oct 23;44(47):4615–25. doi: 10.1038/s41388-025-03608-5 (PMC12623243; doi:10.1038/s41388-025-03608-5)
Supplement: Supplementary file 1 — Legends of Supplementary Figures and Tables [file 41388_2025_3608_MOESM1_ESM.pdf]

Legends of supplemental figures and tables

Supplemental Figure S1: H&E stained section of Merkel cell carcinoma lesion #1 exhibiting epidermotropism. Red frame indicates the selected area of the tissue that was sequenced with high-resolution spatial transcriptomics.

Supplemental Figure S2: H&E stained section of Merkel cell carcinoma lesion #2 exhibiting epidermotropism. Red frame indicates the selected area of the tissue that was sequenced with high-resolution spatial transcriptomics.

Supplemental Figure S3: H&E stained section of Merkel cell carcinoma lesion #3 exhibiting epidermotropism. Red frame indicates the selected area of the tissue that was sequenced with high-resolution spatial transcriptomics.

Supplemental Figure S4: H&E stained section of Merkel cell carcinoma lesion #4 exhibiting epidermotropism. Red frame indicates the selected area of the tissue that was sequenced with high-resolution spatial transcriptomics.

Supplemental Figure S5: H&E stained section of a Merkel cell carcinoma lesion without epidermal involvement used in Figure 2E-F (Ctrl). Red frame indicates the area that was sequenced with spot-based Visium Spatial transcriptomics.

Supplemental Figure S6: Graph-based clustering and spatial mapping of gene expression in sample #1 at 8 µm bin resolution. Spatial transcriptomic data were aggregated into 8 µm bins, followed by unsupervised graph-based clustering. Cluster assignments were then reprojected onto the original tissue coordinates to visualize spatial gene expression patterns.

Supplemental Figure S7: Graph-based clustering and spatial mapping of gene expression in sample #2 at 8 µm bin resolution. Spatial transcriptomic data were aggregated into 8 µm bins, followed by unsupervised graph-based clustering. Cluster

assignments were then reprojected onto the original tissue coordinates to visualize spatial gene expression patterns.

Supplemental Figure S8: Graph-based clustering and spatial mapping of gene expression in sample #3 at 8 µm bin resolution. Spatial transcriptomic data were aggregated into 8 µm bins, followed by unsupervised graph-based clustering. Cluster assignments were then reprojected onto the original tissue coordinates to visualize spatial gene expression patterns.

Supplemental Figure S9: Graph-based clustering and spatial mapping of gene expression in sample #4 at 8 µm bin resolution. Spatial transcriptomic data were aggregated into 8 µm bins, followed by unsupervised graph-based clustering. Cluster assignments were then reprojected onto the original tissue coordinates to visualize spatial gene expression patterns.

Supplemental Figure S10: Distributions of the number of bins, number of expressed genes and UMI counts per segmented cell in samples #1 to #4 after quality filtering.

Supplemental Figure S11: Impact of *TAP63* overexpression in MCC cell line WaGa. (A) Relative mRNA expression of *PERP*, *TP53*, *TAP63* and *ΔNP63* in 5 MCPyV-positive (WaGa, MKL-1, MKL-2, UM-MCC002, UM-MCC005) and one MCPyV-negative (UM-MCC034) MCC cell lines using *RPL10A* as reference gene. Values are in mean ± S.E.M. of 3 independent biological replicates; average Cq values of target genes are shown in Supplemental Table S6. **(B)** GFP and mCherry expression in WaGa cells transduced with the lentiviral vector pLV[Exp]-EGFP/Puro-EF1A>mCherry, under control of the EF1A promoter (upper row). GFP expression in WaGa cells overexpressing *TAP63*, transduced with the lentiviral vector pLV[Exp]-EF1A>hTP63ORF003769:P2A:EGFP:T2A:Puro under control of the EF1A promoter (lower row). **(C)** mRNA expression of *TAP63* in wild-type (WT), mock-transfected control, and *TAP63*-overexpressing (TAP63-OE) WaGa MCC cells, as measured by quantitative PCR (qPCR) and depicted as Ct values. Data are presented as mean ± SEM

from 3 independent biological replicates. **(D)** mRNA expression of *RPL10A, PERP*, and *TAP63* in wild-type (WT), mock-transfected control, and *TAP63*-overexpressing (TAP63-OE) WaGa MCC cells as assessed by reverse transcription PCR. PCR products were separated by agarose gel electrophoresis and visualized using SYBR™ Safe DNA Gel Stain. A 100 bp Plus DNA ladder was used as a molecular size marker. **(E)** Relative mRNA expression of *ΔNP63* in wild type (WT), mock-transfected (control), and *TAP63*-overexpressing (TAP63-OE) WaGa MCC cells. Gene expression level was normalized to wild type WaGa cells. Values are in mean ± S.E.M. of 3 independent biological replicates; average Cq values of target genes are shown in Supplemental Table S7.

Supplemental Table S1: Comparison of the number of expressed genes and UMI counts between 2µm bin set and segmented cell set.

Supplemental Table S2: Feature loading scores of the first 5 components of the harmonized principal component analysis on the segmented cells.

Supplemental Table S3: Differentially expressed genes between interfollicular and follicular infundibulum epiMCC, as well as cMCC/vasMCC from validation single-cell RNA data.

Supplemental Table S4: Differential analysis result of 135 transcriptional regulons with significant differential activities between epiMCC and cMCC+vasMCC from validation single-cell RNA data using Wilcoxon test.

Supplemental Table S5: Functional enrichment analysis of the 114 upregulated genes by enhanced transcription factors in epiMCC from validation single-cell RNA data using gprofiler2.

Supplemental Table S6: Average Cq values of *PERP*, *TP53*, *ΔNP63, TAP63,* and the reference gene *RPL10A* in in 5 MCPyV-positive (WaGa, MKL-1, MKL-2, UM-MCC002, UM-MCC005) and one MCPyV-negative (UM-MCC034) MCC cell lines.

Supplemental Table S7: Average Cq values of *PERP*, *TP53*, *ΔNP63, TAP63*, and reference gene *RPL10A* in WaGa cells with *TAP63* overexpression (TAP63-OE), with empty vector (Control) and untransfected wildtype (WT).

Supplemental Table S8: Primer sequences used to perform qPCR.
