# Supplementary material for: High-resolution spatial transcriptomics uncover epidermal-dermal divergences in Merkel cell carcinoma: spatial context reshapes the gene expression landscape
Source: Oncogene. 2025 Oct 23;44(47):4615–25. doi: 10.1038/s41388-025-03608-5 (PMC12623243; doi:10.1038/s41388-025-03608-5)
Supplement: Supplementary file 2 — Supplementary Information [file 41388_2025_3608_MOESM2_ESM.pdf]

Supplemental Methods

High-definition spatial transcriptomics library construction

Tissue preparation and library construction for Visium Spatial HD sequencing were performed in accordance with the protocols provided by 10x Genomics (Visium HD Human transcriptome 6.5mm, PN-1000675, 10x Genomics, Leiden, The Netherlands). Briefly, archived FFPE slides were deparaffinized and stained with H&E (H&E Staining Kit ab245880, Abcam), after which the stained slides were scanned with 3DHistech Pannoramic MIDI (Epredia/Microm International GmbH, Dreieich, Germany). After the slides were destained and decrosslinked, probe hybridization and ligation were performed with Visium Human Transcriptome Probe set v2.0, followed by probe release where single-stranded ligation products were released from the tissue and captured on the Visium HD slide with Visium CytAssist instrument (10X Genomics). This was followed by extension twice to capture maximum probes as possible and the extended ligation products with unique molecular identifiers and spatial barcodes were eluted from Visium HD slides, followed by amplification, quantification and sample indexing to generate final library molecules as specified in the standard 10x protocols for library construction. Visium Spatial FFPE sequencing was also performed as previously described (ref. 15). Libraries were sequenced at the NGS Core Facility at DKFZ, Heidelberg, with Illumina NovaSeq 6000. Sequenced probe reads were analyzed using 10x Space Ranger 3.0.0 with the precompiled GRCh38-2020 human reference genome. To facilitate cell segmentation in the following step, we trimmed the original H&E images to reduce the size, allowing a 2% margin surrounding the capture area.

Spot-based spatial transcriptomics library construction

Archived specimen of an MCC lesion without epidermal involvement was processed following the protocols for Visium CytAssist Spatial Gene Expression for FFPE from 10X Genomics

(Visium CytAssist Spatial Gene and Protein Expression, CG000659, 10x Genomics). Briefly, FFPE section on the slide was deparaffinized, stained with H&E (H&E Staining Kit ab245880, Abcam) and imaged with 3DHistech Pannoramic MIDI (Epredia/Microm International GmbH). After de-crosslinking the stains, probe hybridization followed by ligation was performed with human whole transcriptome probes v2. The slide was then loaded onto Visium CytAssist instrument (10x Genomics) to transfer the FFPE section onto Visium CytAssist Spatial Gene Expression slide, which was then processed following standard protocols for library construction. The library was then sent to NGS Core Facility in DKFZ, Heidelberg for sequencing. The sequenced probe reads were analyzed using 10X Space Ranger 2.0.0 with precompiled GRCh38-2020 human reference genome and Visium Human Transcriptome Probe set v2.0 provided by 10X Genomics. High-resolution whole slide scan of H&E-stained section are depicted in Supplemental Figure S5.

Cell segmentation

Cell segmentation was performed using bin2cell v.0.1.0 on Python 3.10.14 (ref. 16) with default parameters. Briefly, for each sample, the trimmed H&E image, rescaled to 0.5 micron per pixel (mpp), was used to run StarDist with pretrained 2D_versatile_he model. 2-micron unique molecular identifier (UMI) count matrix from Space Ranger output was imported and filtered to include genes found in ≥ 3 tiles. The filtered expression matrix was de-striped, the NaN (i.e. Not a Number) values were set to zero, and then the real-numbered count matrix was rounded down to the nearest integer. The adjusted count matrix was saved as gene expression image with 0.5 mpp and sigma=5 for Gaussian filter. The gene expression image was then used to run StarDist with pretrained 2D_versatile_fluo model. StarDist determined cell masks denoting boundaries of individual cells from H&E and gene expression images separately. H&E cell masks were expanded by a maximum of 2 tiles if no other cell masks were present in the surrounding. Gene expression cell masks were used as secondary means,

to fill gaps but only if there were no overlapping H&E cell masks in the area. Once the cell masks were determined, UMI counts in cell level were quantified.

Quality filtering of the segmented cells

The segmented cells from bin2cell were further filtered to obtain high-confidence cell set in a two-pass approach. Cells which were composed of less than 5 or more than 50 tiles were removed as they were likely artifacts or multiplets. After the first pass, the remaining cells were clustered with the R package Seurat v5.2.1. Briefly, the counts were log-normalized, top 2000 highly variable genes were identified and scaled. Principal component analysis (PCA) was performed and the top 10 principal components were used to perform Uniform Manifold Approximation and Projection (UMAP) dimensionality reduction as well as shared nearest neighborhood clustering, with resolution set to 0.2. Clusters that had low gene/UMI counts or reflected sequencing artifact (e.g. frame of tissue slide cassette) were removed. Cells remaining after the second pass were used for downstream analyses.

Manual selection of segmented cells

We used the python package napari v.0.4.19.post1 to choose the cells of interest in an interactive manner. The segmented cell object from bin2cell pipeline (i.e. output from subsection "Cell segmentation") was imported into the Python environment with read_h5ad function from Python package Scanpy v.1.9.8 and only the cells passing quality check from previous section were retained. The filtered data along with H&E image was visualized in napari viewer, where the coordinate and index of each cell were overlaid on the H&E image. By hovering the mouse cursor over a nucleus in the H&E image, the corresponding segmented cell coordinates were displayed and recorded. MCC cells from both epidermal regions (interfollicular and follicular infundibulum) and dermal regions were identified based on morphological criteria (ref. 17), as well as information from sequential CK20-stained sections. Basal and suprabasal keratinocytes located at a distance from the tumor were selected as references.

Preprocessing of single-cell RNA sequencing data

The data deposited in NCBI GEO with accession number GSE226438 was fetched for reanalysis. Briefly, raw FastQ files from SRA Run Selector were downloaded, then aligned and quantified using Cell Ranger v.7.1.0 with a modified version of precompiled GRCh38-2020-A human genome provided by 10x Genomics, which incorporated Merkel cell polyomavirus genome obtained from NCBI with accession number NC_010277.2. The output files from Cell Ranger were analyzed with Seurat. Cells with less than 200 expressed genes, higher than 30% mitochondrial gene expression or 50% hemoglobin gene expression (i.e. *HBA1, HBA2, HBB*) were removed. The filtered cells were log-normalized and cell cycle score was calculated with predefined gene sets from Seurat, then the expression was renormalized using SCTransform while regressing out mitochondrial gene percentage and cell cycle score difference. The first 30 principal components were used to perform shared nearest neighborhood clustering. Finally, individual cell sets were merged and renormalized with SCTransform with regression on mitochondrial gene percentage and cell cycle difference.

TAp63 overexpression in MCC Cells

A recombinant lentiviral vector expressing human TP63 [ORF003769], tagged with EGFP (pLV[Exp]-EF1A>hTP63ORF003769:P2A:EGFP:T2A:Puro), and a control vector expressing mCherry (pLV[Exp]-EGFP/Puro-EF1A>mCherry) were generated by VectorBuilder (Cat. No. VB250505-1052ncn and VB010000-9492agg respectively). Corresponding ultra-purified recombinant lentiviral particles were produced by the same provider (VectorBuilder, Inc., Cat. No.: LVI(VB250505-1052ncn)-K1, Lot 250520LVH51).

For transduction, WaGa cells were incubated with viral supernatant and 1 µg/mL polybrene (VectorBuilder, Inc, Cat. No. PL200) for 4 hours, followed by 2 washes with fresh culture medium. Transduction efficiency was evaluated in the following days using the Zeiss Axio Observer by evaluating the ratio of EGFP-positive cells in the experimental cells and EGFP and mCherry-positive cells in the control group (see Supplementary Figure S11B). To select

transduced cells, 1 µg/mL puromycin (Thermo Fisher Scientific, Cat. No. A1113803) was applied for 4 days.

Quantitative Real-Time Reverse Transcriptase PCR analysis

Total RNA was extracted using the RNeasy Plus Mini Kit (Qiagen GmbH, Cat. No. 7413654) followed by treatment with recombinant DNase I (Sigma-Aldrich, Cat. No. 471672800). cDNA synthesis was performed using SuperScript IV Reverse Transcriptase (Thermo Fisher Scientific, Cat. No. 8090200) according to the manufacturer's instructions. Quantitative real-time PCR was conducted using the CFX Real-Time PCR System (Bio-Rad Laboratories, Düsseldorf, Germany). mRNA levels of *TP53*, *PERP*, *TAP63*, and *ΔNP63* were quantified using Luna Universal qPCR Master Mix (New England Biolabs, Cat. No. M3003E) and the respective primers. RPLP10A served as a reference gene. Primer sequences are provided in Supplemental Table S8 (ref. 18). PCR efficiency was determined using external standards on plasmid mini-preparation of cloned PCR products. Expression levels were analyzed by basic relative quantification. Relative expression levels were calculated using the $2^{-\Delta\Delta Cq}$ method. qPCR data were based on 2 to 4 biological replicates, with 3 technical replicates for each biological replicate. ΔΔCq and Cq values are provided in Supplemental Table S6 and S7.

Bioinformatics analysis

Differential gene expression analysis of single-cell RNA sequencing data was conducted using the Wilcoxon rank-sum test implemented in Seurat. Genes were considered significantly differentially expressed if their Bonferroni-adjusted p-values were below 0.05 and they exhibited a minimum expression in at least 40% of cells in either group.

Transcriptional factor activity inference was performed with R package decoupleR v.2.8.0. CollecTRI, a curated database of transcription factor-target interactions, was used for activity inference. Target genes regulated by activated transcription factors were selected based on criteria of enhanced transcription factor activity, defined as a mean $\log_2$ fold change > 2 and

an adjusted p-value < 0.05 in the Wilcoxon rank-sum test. SCT-normalized expression of single-cell RNA data was used to compute activity scores with univariate linear model as specified in decoupleR protocol. The activity scores per cell were scaled and differentially activated regulons were identified with Wilcoxon rank-sum test.

Functional analysis of upregulated transcription factor regulon genes was performed with Cytoscape v.3.10.1. A target gene network was constructed using GeneMANIA v.3.5.2, then the enriched pathways of the network were computed using EnrichmentTable v.2.0.5. A cutoff of 0.5 was set to remove functional terms that shared 50% of their genes to reduce redundancy.

Gene set enrichment analysis for single-cell RNA sequencing data was conducted with the R package fgsea v.1.32.2 on differentially expressed genes ranked by $\log_2$ fold change. Gene sets corresponding to Gene Ontology (GO) biological processes were obtained from the C5 collection (version c5.go.bp.v2023.1.Hs) of the Molecular Signature Database (MSigDB).

Gene set activity analysis was performed with the R package AUCell v.1.28.0 on SCT-normalized expression from both combined segmented data cells and single-cell RNA sequencing data. Tumor transcriptional metaprogram gene sets were generated based on Gavish et al. as previously described (ref. 19).

Visualization of significance in violin plots was done with R package ggsignif v.0.6.4 with Wilcoxon rank-sum test in default parameters. P-values were unadjusted. ***P<0.001, **P<0.01, *P<0.05, NS=not significant.

Supplemental Figure S1

#1

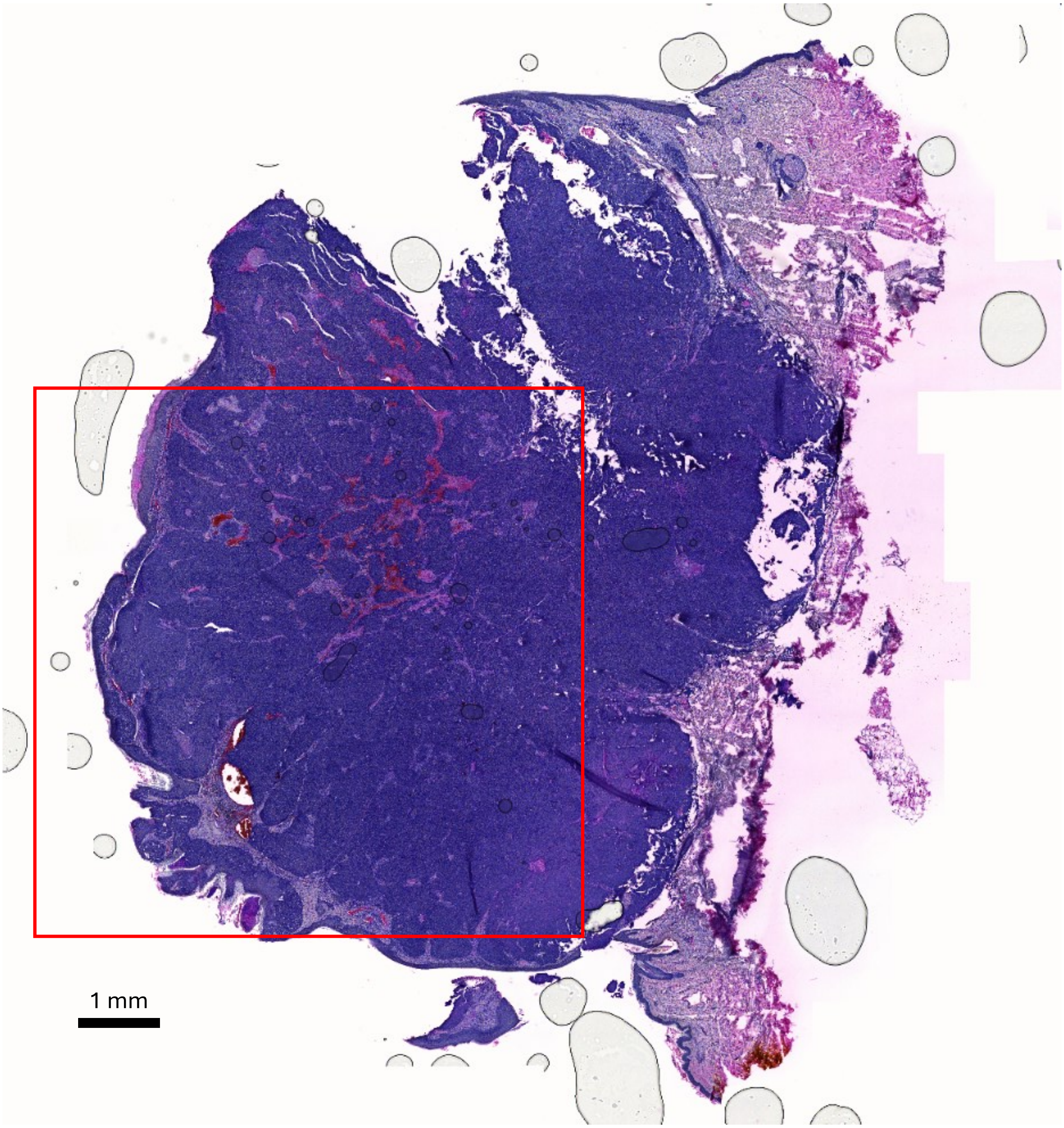

Supplemental Figure S1: H&E stained section of Merkel cell carcinoma lesion #1 exhibiting epidermotropism. Red frame indicates the selected area of the tissue that was sequenced with high-resolution spatial transcriptomics.

Supplemental Figure S2

#2

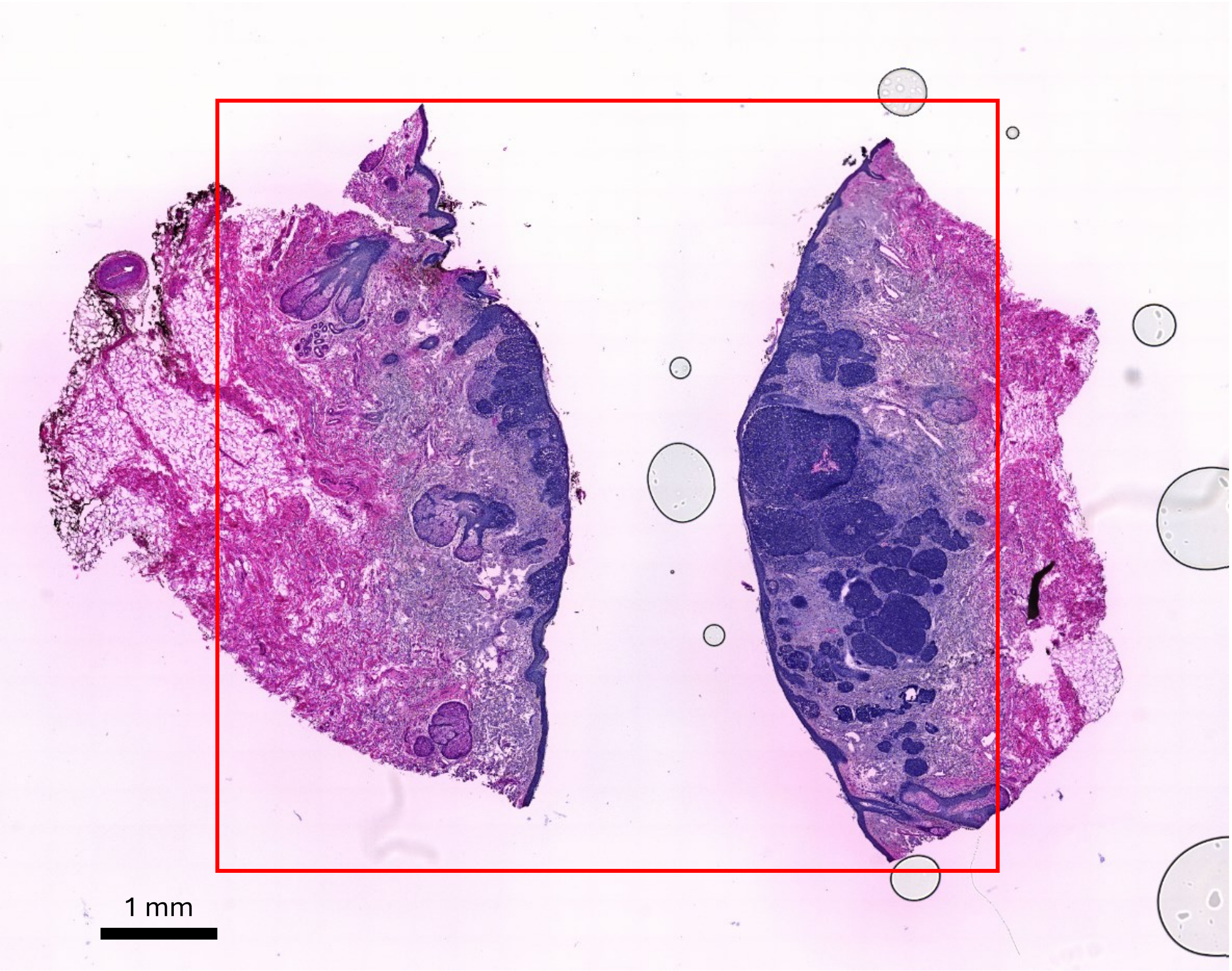

Supplemental Figure S2: H&E stained section of Merkel cell carcinoma lesion #2 exhibiting epidermotropism. Red frame indicates the selected area of the tissue that was sequenced with high-resolution spatial transcriptomics.

Supplemental Figure S3

#3

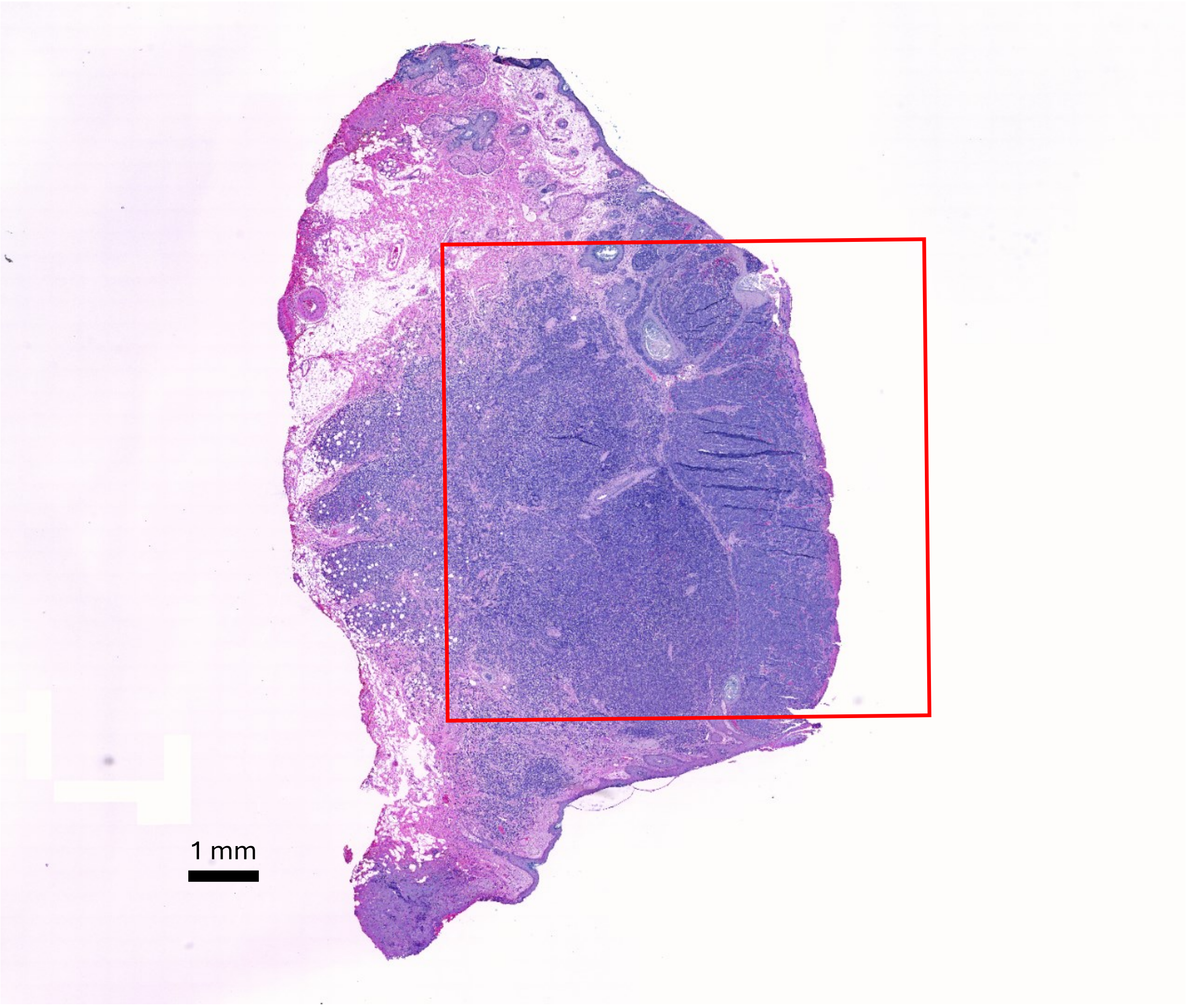

Supplemental Figure S3: H&E stained section of Merkel cell carcinoma lesion #3 exhibiting epidermotropism. Red frame indicates the selected area of the tissue that was sequenced with high-resolution spatial transcriptomics.

Supplemental Figure S4

#4

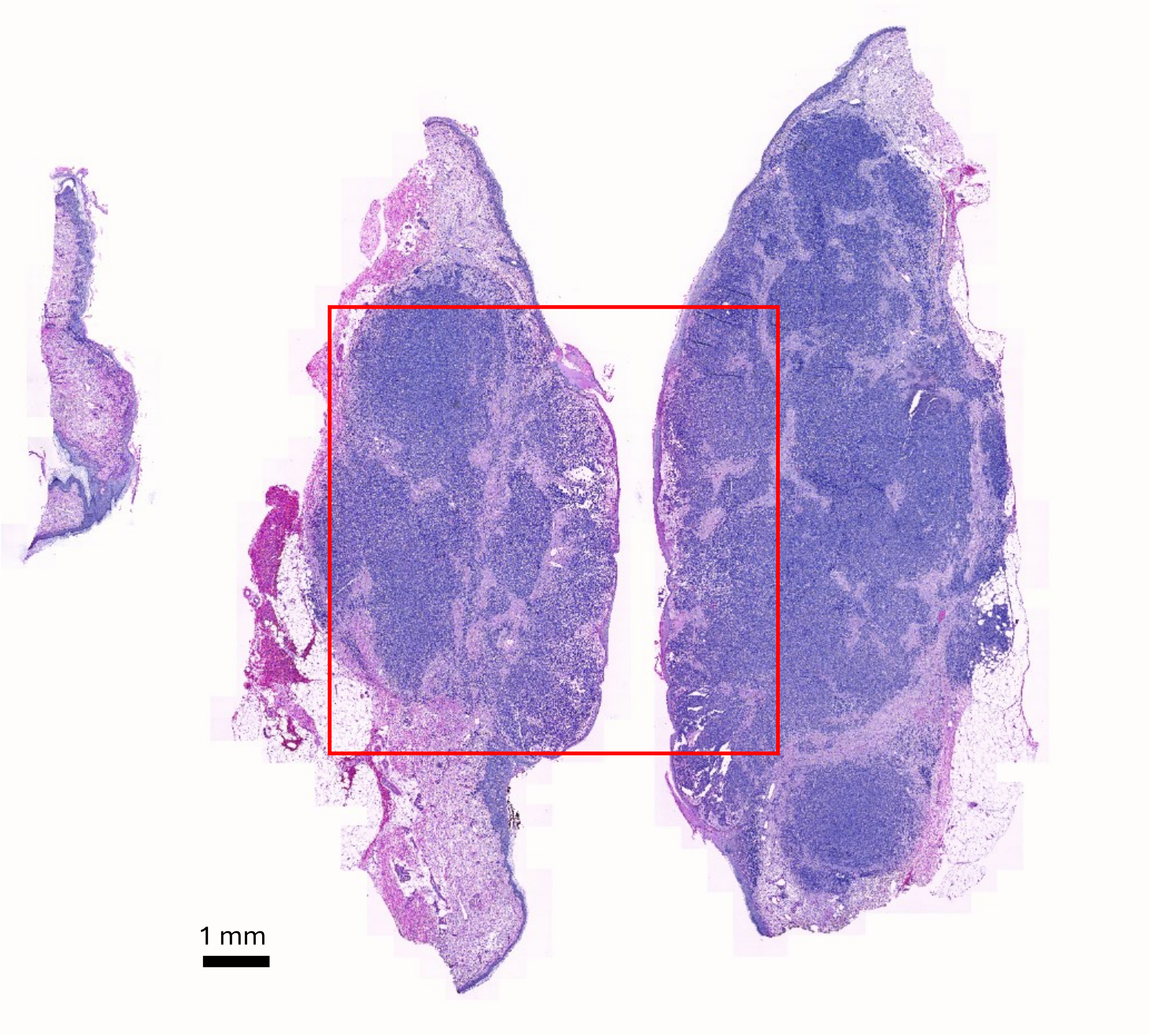

Supplemental Figure S4: H&E stained section of Merkel cell carcinoma lesion #4 exhibiting epidermotropism. Red frame indicates the selected area of the tissue that was sequenced with high-resolution spatial transcriptomics.

Supplemental Figure S5

Ctrl

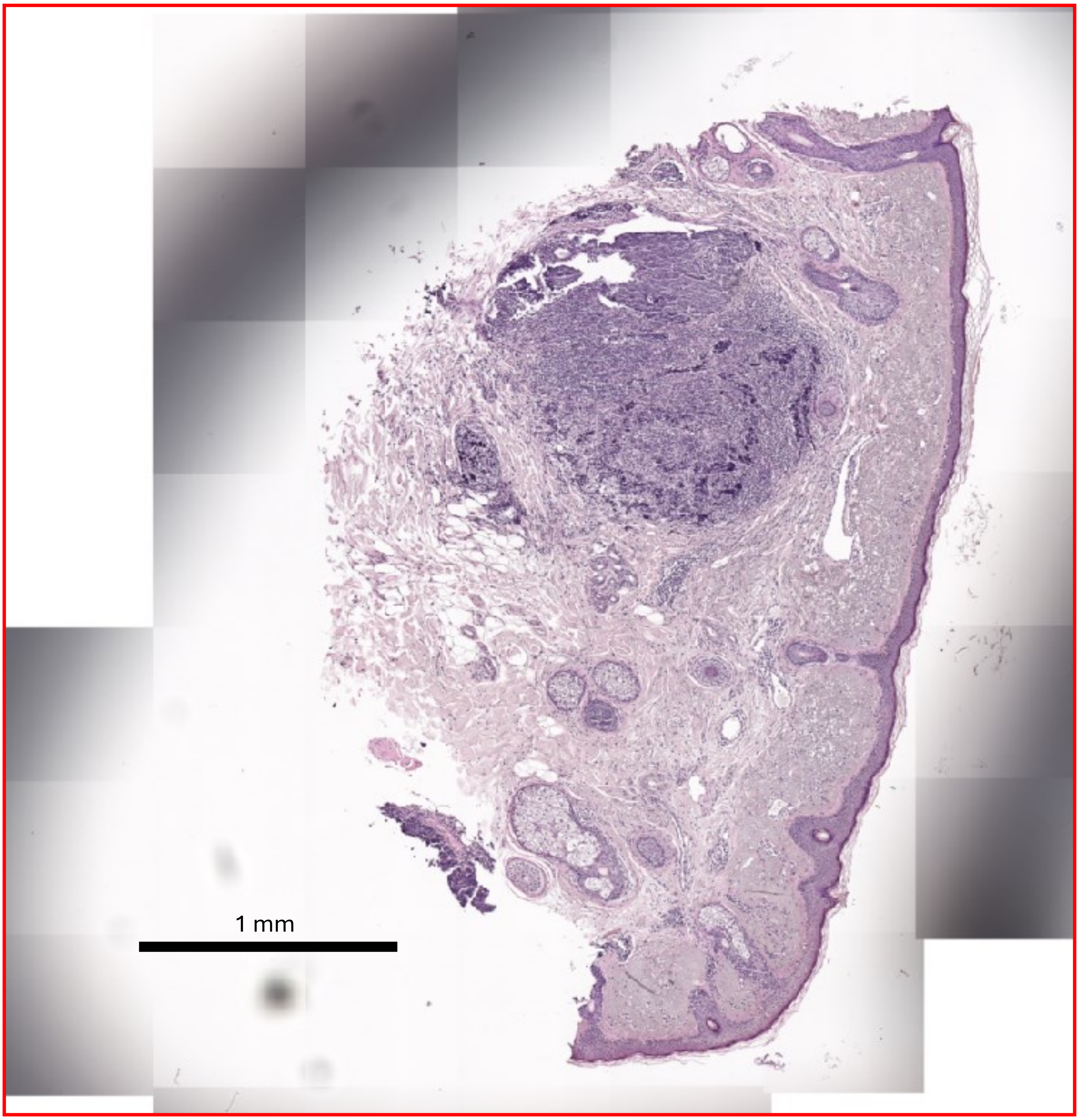

Supplemental Figure S5: H&E stained section of a Merkel cell carcinoma lesion without epidermal involvement used in Figure 2E-F (Ctrl). Red frame indicates the area that was sequenced with spot-based Visium Spatial transcriptomics.

Supplemental Figure S6

#1

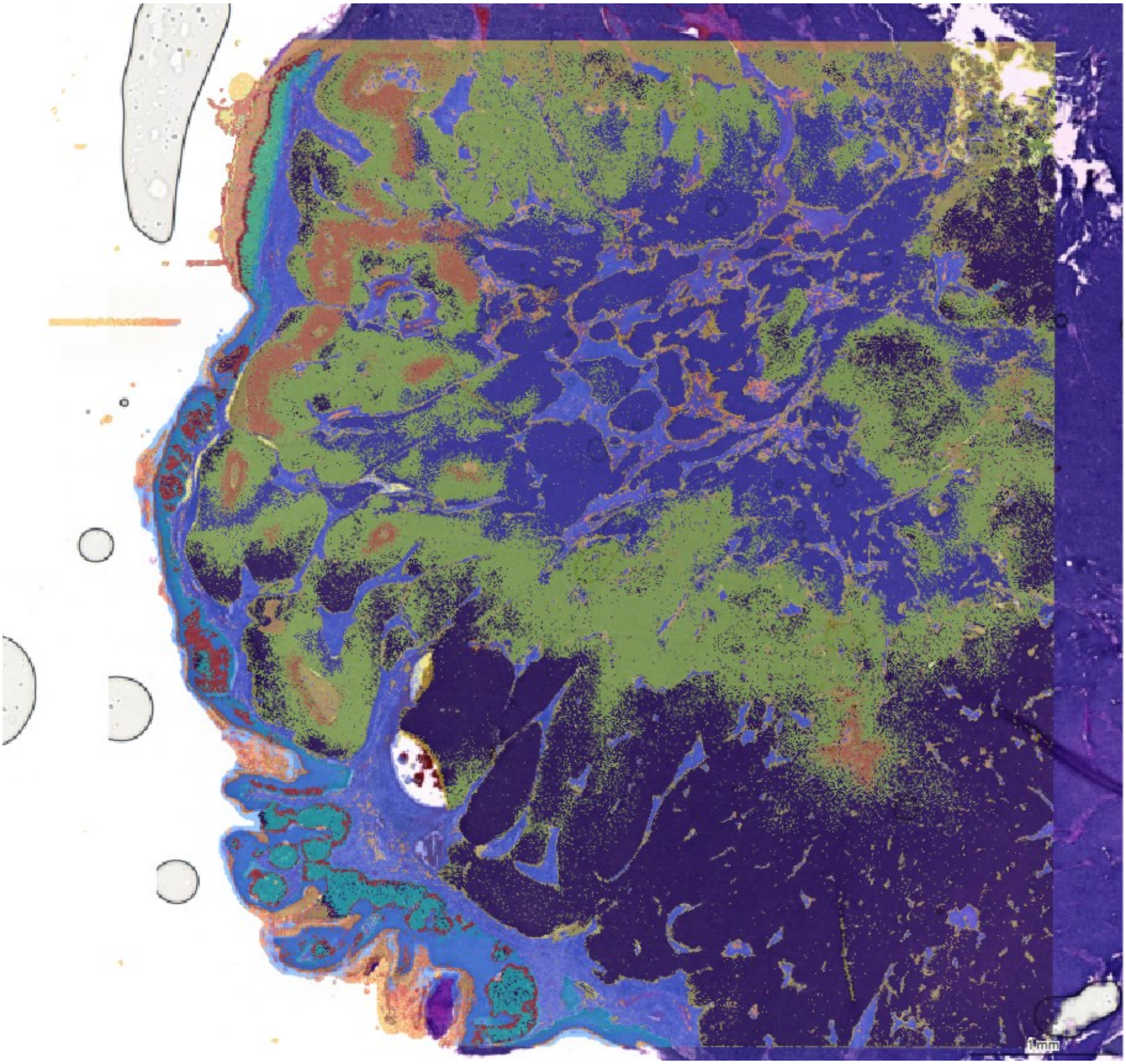

Supplemental Figure S6: Graph-based clustering and spatial mapping of gene expression in sample #1 at 8 μm bin resolution. Spatial transcriptomic data were aggregated into 8 μm bins, followed by unsupervised graph-based clustering. Cluster assignments were then reprojected onto the original tissue coordinates to visualize spatial gene expression patterns.

Supplemental Figure S7

#2

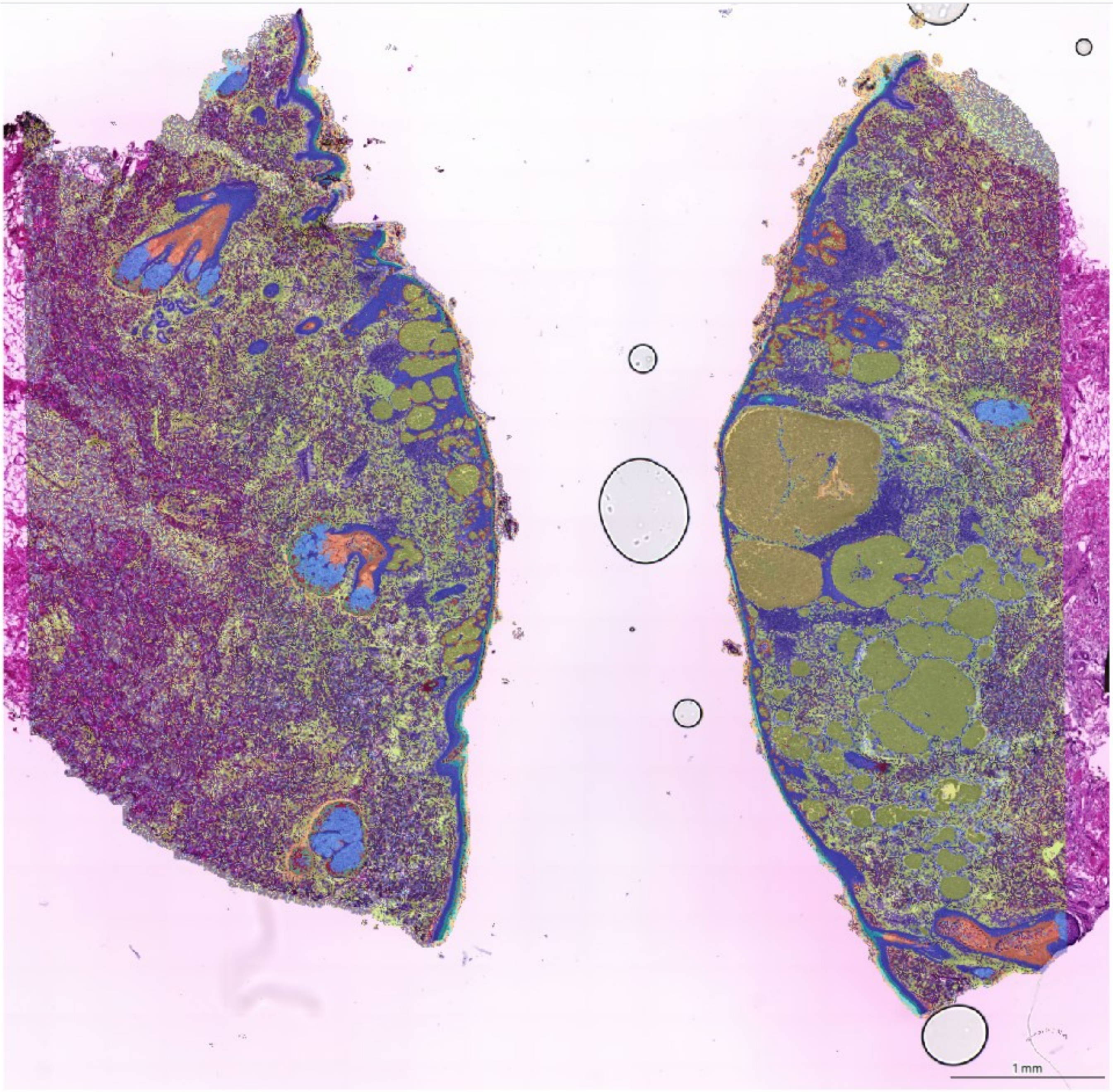

Supplemental Figure S7: Graph-based clustering and spatial mapping of gene expression in sample #2 at 8 µm bin resolution. Spatial transcriptomic data were aggregated into 8 µm bins, followed by unsupervised graph-based clustering. Cluster assignments were then reprojected onto the original tissue coordinates to visualize spatial gene expression patterns.

Supplemental Figure S8

#3

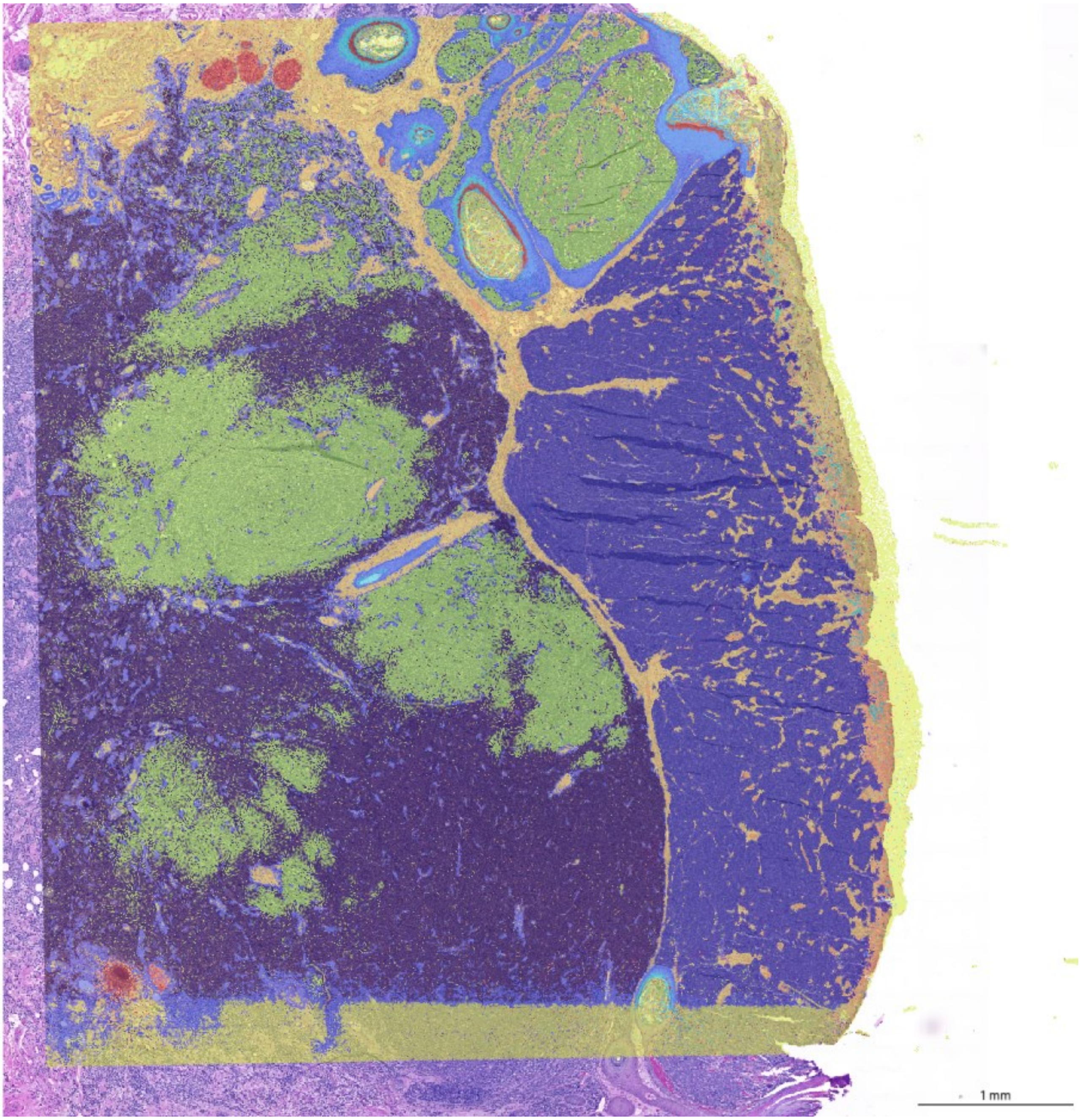

Supplemental Figure S8: Graph-based clustering and spatial mapping of gene expression in sample #3 at 8 µm bin resolution. Spatial transcriptomic data were aggregated into 8 µm bins, followed by unsupervised graph-based clustering. Cluster assignments were then reprojected onto the original tissue coordinates to visualize spatial gene expression patterns.

Supplemental Figure S9

#4

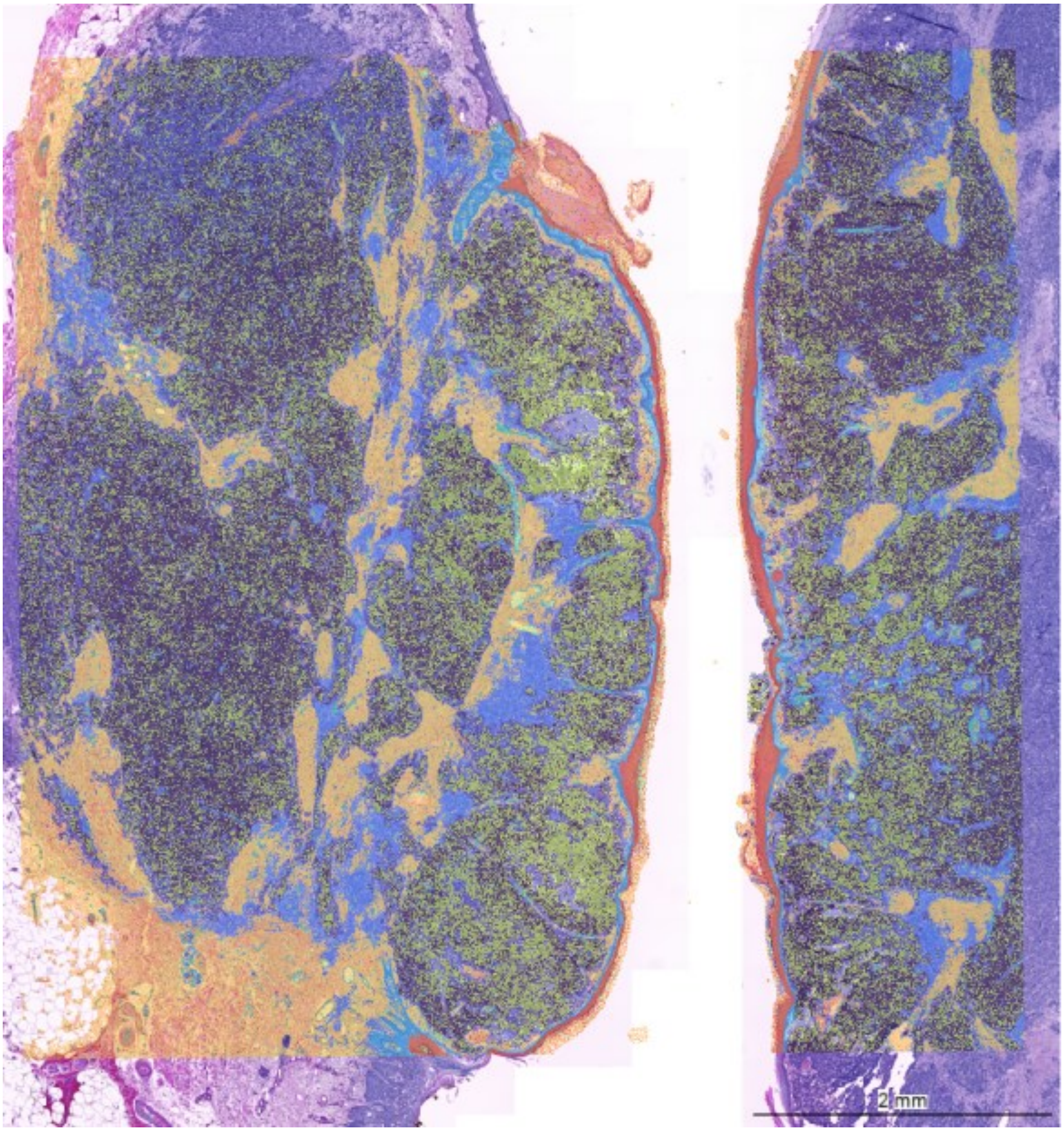

Supplemental Figure S9: Graph-based clustering and spatial mapping of gene expression in sample #4 at 8 µm bin resolution. Spatial transcriptomic data were aggregated into 8 µm bins, followed by unsupervised graph-based clustering. Cluster assignments were then reprojected onto the original tissue coordinates to visualize spatial gene expression patterns.

Supplemental Figure S10

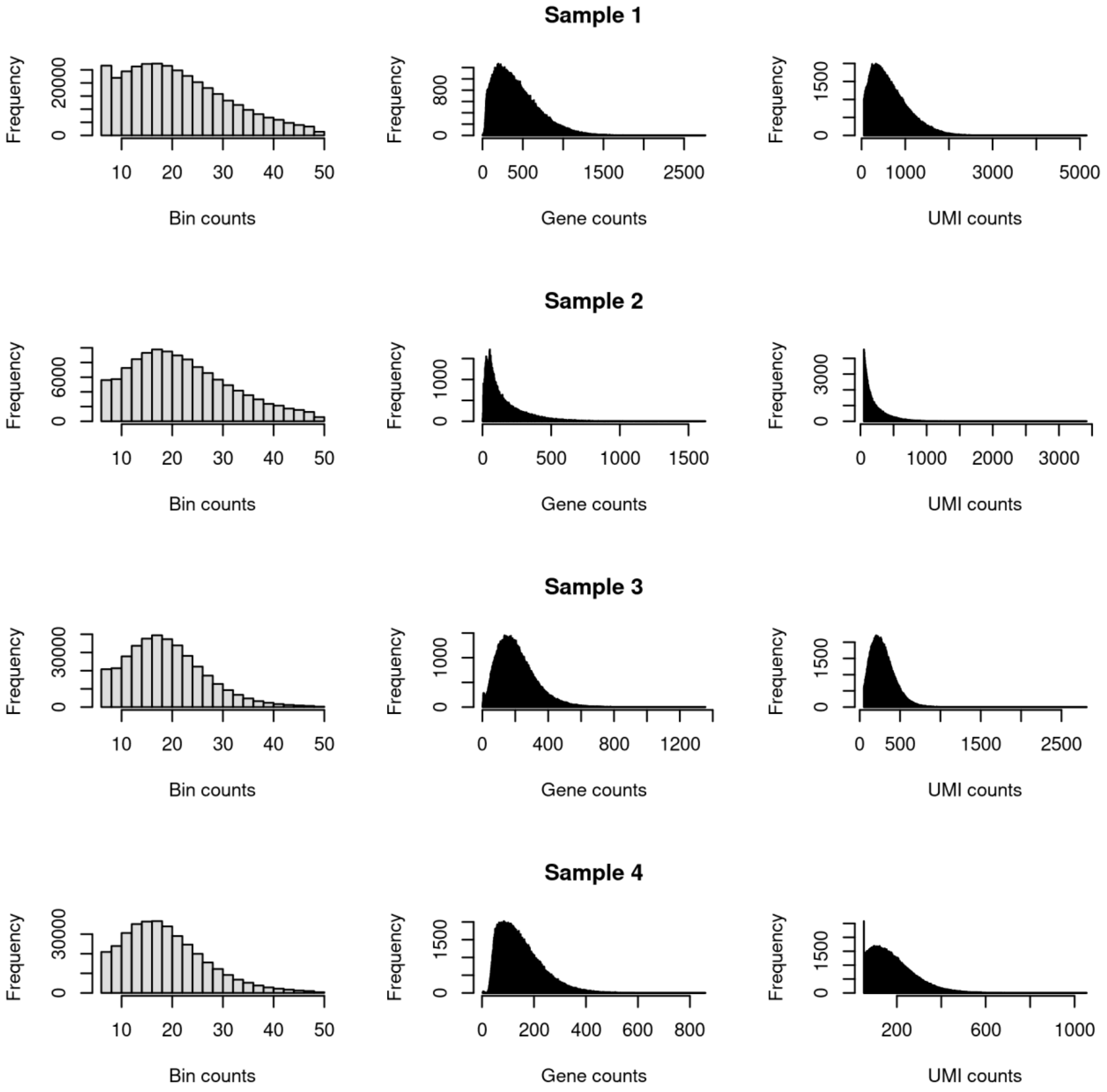

Supplemental Figure S10: Distributions of the number of bins, number of expressed genes and UMI counts per segmented cell in samples #1 to #4 after quality filtering.

Supplemental Figure S11

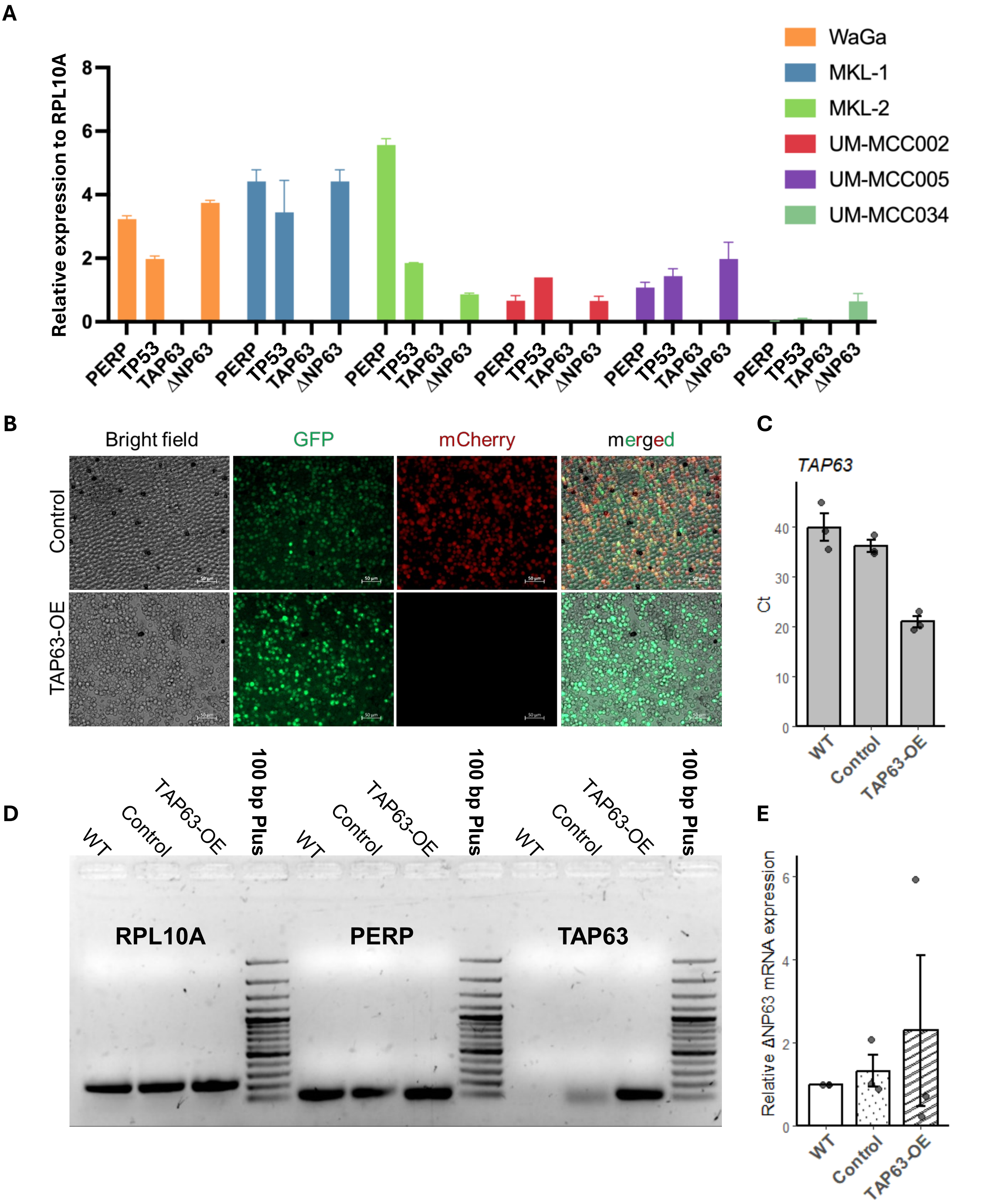

Supplemental Figure S11: Impact of *TAP63* overexpression in MCC cell line WaGa. (A) Relative mRNA expression of *PERP*, *TP53*, *TAP63* and *ΔNP63* in 5 MCPyV-positive (WaGa, MKL-1, MKL-2, UM-MCC002, UM-MCC005) and one MCPyV-negative (UM-MCC034) MCC cell lines using *RPL10A* as reference gene. Values are in mean ± S.E.M. of 3 independent biological replicates; average Cq values of target genes are shown in Supplemental Table S6. **(B)** GFP and mCherry expression in WaGa cells transduced with the lentiviral vector pLV[Exp]-EGFP/Puro-EF1A>mCherry, under control of the EF1A promoter (upper row). GFP expression in WaGa cells overexpressing *TAP63*, transduced with the lentiviral vector pLV[Exp]-EF1A>hTP63ORF003769:P2A:EGFP:T2A:Puro under control of the EF1A promoter (lower row). **(C)** mRNA expression of *TAP63* in wild-type (WT), mock-transfected control, and *TAP63*-overexpressing (TAP63-OE) WaGa MCC cells, as measured by quantitative PCR (qPCR) and depicted as Ct values. Data are presented as mean ± SEM from 3 independent biological replicates. **(D)** mRNA expression of *RPL10A*, *PERP*, and *TAP63* in wild-type (WT), mock-transfected control, and *TAP63*-overexpressing (TAP63-OE) WaGa MCC cells as assessed by reverse transcription PCR. PCR products were separated by agarose gel electrophoresis and visualized using SYBR™ Safe DNA Gel Stain. A 100 bp Plus DNA ladder was used as a molecular size marker. **(E)** Relative mRNA expression of *ΔNP63* in wild type (WT), mock-transfected (control), and *TAP63*-overexpressing (TAP63-OE) WaGa MCC cells. Gene expression level was normalized to wild type WaGa cells. Values are in mean ± S.E.M. of 3 independent biological replicates; average Cq values of target genes are shown in Supplemental Table S7.
